# Genome Assembly of *Arctica islandica*, the Longest-Lived Non-Colonial Animal Species

**DOI:** 10.3390/ani15050690

**Published:** 2025-02-27

**Authors:** Glenn S. Gerhard, John B. Allard, Scott Kaniper, Dorret Lynch, Hayan Lee, Sudhir Kumar

**Affiliations:** 1 Department of Medical Genetics and Molecular Biochemistry, Lewis Katz School of Medicine at Temple University, Philadelphia, PA 19140, USA; skaniper@temple.edu (S.K.); lynchd@temple.edu (D.L.); 2 Institute for Genomics and Evolutionary Medicine, Temple University, Philadelphia, PA 19122, USA; john.allard@temple.edu (J.B.A.); s.kumar@temple.edu (S.K.); 3 Department of Biology, Temple University, Philadelphia, PA 19122, USA; 4 Cancer Epigenetics Institute, Nuclear Dynamics and Cancer Program, Fox Chase Cancer Center, Philadelphia, PA 19111, USA

**Keywords:** *Arctica islandica*, genome, assembly, longevity

## Abstract

We report the first high-quality genome-wide assembly for *Arctica islandica*, the longest-lived non-colonial species, with a reported maximum life span of 507 years. The genome was assembled using short- and long-read DNA sequencing and RNA sequencing of four tissues. All assessment approaches indicated that the assembled genome is complete, contiguous, and accurate. The genome size is estimated at 1781.15 million base pairs (Mbps) with a coverage of 247.8×. The heterozygous rate was 1.15% and the repeat content 67.66%. Genome completeness evaluated by complete BUSCOs was 92.7%. The non-redundant gene set consisted of 39,509 genes with an average transcript length of 15,429 bp. More than 98% of the genes could be annotated across databases. Predicted non-coding RNAs included 801 miRNAs, 11,114 tRNAs, 909 rRNAs, and 349 snRNAs. The *Arctica islandica* genome, along with the assembly of genomes from other clam species, sets the stage for elucidating the molecular basis for the convergence of extreme longevity across these bivalve species.

## Simple Summary

Only a few dozen of the animal species on Earth can live for more than 100 years. The longest-lived animal is the ocean quahog, also known as the mahogany clam, which can live for more than 500 years. We determined the DNA sequence of the ocean quahog genome. Knowing the sequence of the longest-lived animal will allow for the comparison of its genome against long- and short-lived animals to identify the genes and pathways associated with a long life span.

## 1. Introduction

The class Bivalves, which includes clams, has in excess of 10,000 species with a wide diversity of life histories, environments, and longevities [1,2]. They have garnered some interest as models for aging research in part because chronological age for many bivalve species can be accurately estimated using the annual growth rings on their shells, which have been validated using independent techniques [3]. The wide range of maximum longevities that has been observed across bivalve species ranges from about 1 year to over 500 years [4]. Such extreme longevity appears to have evolved independently across diverse animal species, with bivalves well represented [5]. As a group, bivalves may thus be particularly amenable to evolutionary studies of aging. For example, evolutionary approaches to extreme longevity in bony fish species have been reported [6].

For molecular evolutionary studies, high-quality genomic information is needed. In 2010, Philipp and Abele [1] noted, “Genomic sequence information on bivalves is still very limited”. High-quality sequenced genomes of bivalve species are still sparse over a decade later. Here, we report a draft genome sequence of *Arctica islandica* Linnaeus 1767, also called the ocean quahog, mahogany clam, Iceland Cyprina, and mud clam. *Arctica islandica* is known as the most longevous non-colonial species with an average longevity greater than 225 years and one individual (061294) with a reported maximum life span of 507 years (AD 1499–2005) [7]. We compared features of the *Arctica islandica* genome to several other clam species including the long-lived *Panopea generosa* [8], *Margaritifera margaritifera* [9], *Tridacna gigas* [10], and *Mercenaria mercenaria* [11], and the shorter-lived species *Tridacna crocea* [12], *Mactromeris polynyma* [13], *Serripes groenlandicus* [14], and *Ruditapes philippinarum* [15]. The maximum life spans of these species range from less than 10 years to several with longevities of more than 100 years. Such a broad range of life spans in species with similar anatomical and cellular compositions implies that genetic differences may exist underlying their divergence in longevity. The pace at which computational power for comparative analyses is increasing may be outpacing the availability of high-quality genomic data for analysis. While bivalves, such as *Arctica islandica* and other long-lived clam species, have great potential as models for longevity studies, the current lack of high-quality genomic information limits the in-depth understanding of their aging mechanisms. Thus, this study fills an important knowledge gap by generating a high-quality genome assembly and annotation, providing a valuable resource for future aging and longevity research.

## 2. Materials and Methods

### 2.1. Sample Collection and Nucleic Acid Extraction

Specimens of *Arctica islandica* (Supplementary Material Figure S1) were obtained from Samuels & Son Seafood Co. (Philadelphia, PA, USA). The animals were placed on ice and dissected, with sections of foot, mantle, digestive gland, and gill tissues immediately frozen in dry ice, then stored at −80 °C until analysis. Total DNA was extracted from gill tissue from a single individual using the Qiagen (Hilden, Germany) DNeasy Blood & Tissue Kit, quantified using Nanodrop D-1000, and stored at −80 °C. RNA was extracted from foot, mantle, digestive gland, and gill tissues using the QiaQuick RNA kit (Qiagen, Hilden, Germany).

### 2.2. DNA and RNA Sequencing

Sequencing was performed at Novogene Co., Ltd. (Beijing, China). Short-read Illumina DNA sequencing was performed as described in Supplementary Material Method S1 and Figure S3. Genomic DNA was sheared using a Megaruptor (Liege, Belgium). The fragments were then end-repaired, A-tailed, ligated with Illumina adapters, PCR amplified, size selected, and purified using the Rapid Plus DNA Lib Prep Kit (Illumina, San Diego, CA, USA) for Qubit and real-time PCR quantification and bioanalyzer sizing prior to sequencing on a Novoseq 6000 (Illumina, San Diego, CA, USA). Sequencing quality was assessed by quality Phred score by base position along a read, distribution of error rate along reads, and base distribution along reads. Raw reads were also quality filtered by removing reads containing adapters, more than 10% of uncalled bases, and those with a Q score ≤ 5.

Long-read PacBio DNA sequencing was performed by constructing SMRTbell libraries (Supplementary Material Method S2) using the SMRTbell™ Express Template Prep Kit 2.0 (Pacific Biosciences/PacBio of California, Inc., Menlo Park, CA, USA) following PacBio library instructions. As shown in the workflow in Figure S8, genomic DNA was sheared using a Megaruptor (Liege, Belgium), purified, and end-repaired. After A-tailing, DNA fragments were ligated to the hairpin adaptors, then heat-killed. Damaged or non-intact SMRTbell templates were removed by nuclease treatment, and the remaining products were purified. After size selection, SMRTbell libraries were purified and sequenced in zero-mode waveguides of SMRT^®^ Cells by a PacBio (Menlo Park, CA, USA) Sequel II using the standard protocol of the PacBio Sequel II binding kit 2.0 (Menlo Park, CA, USA) and PacBio Sequel II sequencing kit 2.0 (Menlo Park, CA, USA) to generate data.

Short-read Illumina RNA sequencing was performed as described in Supplementary Material Method S3. Total RNA was extracted from foot, mantle, digestive gland, and gill tissues using the QiaQuick RNA kit (Qiagen, Hilden, Germany). Messenger RNA was purified from total RNA using poly-T oligo-attached magnetic beads. After fragmentation using a Megaruptor (Liege, Belgium), first-strand cDNA was synthesized using random hexamer primers followed by second-strand cDNA synthesis. The library was prepared (Figure S14) using the Fast RNA-seq Lib Prep Kit V2 for end-repair, A-tailing, and adapter ligation. After size selection, amplification, and purification, quantified libraries were pooled and sequenced on a Novoseq 6000 (Illumina, San Diego, CA, USA).

### 2.3. Primary Genome Assembly and Assessment

Before assembly, genome size was estimated by kmer analysis using Kmer = 17 (Supplementary Material Method S4). The overall strategy for genome assembly is described in Supplementary Material Method S5 and Figure S32. Falcon was used for primary genome assembly. PacBio data were self-corrected by read alignment and insertions, deletions, and error to generate pre-assembled reads. After self-correction, the reads were joined using the Overlap–Layout–Consensus algorithm to generate consensus sequences. Genomic error correction was then performed with nextpolish [16] in combination with Illumina short reads and PacBio CLR reads. The genomic sequences were then de-hybridized using purge_haplotigs to further enhance accuracy.

Genome completeness was evaluated by combining BUSCO (Benchmarking Universal Single-Copy Orthologs, http://busco.ezlab.org/) with tools including tblastn, augustus, and hmmer. Genome completeness was also evaluated by combining CEGMA (Core Eukaryotic Genes Mapping Approach, https://github.com/KorfLab/CEGMA_v2), which selected 248 conserved genes in 6 eukaryotes as core genes, with tools including tblastn, genewise, and geneid. Illumina short reads were mapped to the assembled genome by BWA (http://bio-bwa.sourceforge.net/) and the mapping rate/coverage/depth assessed. For the assembled genome, 10k sliding windows were used to calculate GC contents and average sequencing depth to check for possible GC bias contamination during sequencing using Blobtools (v.1.1.1) [17]. Single nucleotide polymorphisms (SNP), i.e., single nucleotide variation, were used along with tools including SAMTools (http://samtools.sourceforge.net/) to order and deduplicate BWA mapping results, call SNPs, and filter original results.

### 2.4. Genome Annotation

Genome annotation (Supplementary Material Method S6) included analysis of repeat sequences, gene structure and gene function, and non-coding RNA (ncRNA) annotation.

For repeat annotation, a combined strategy based on homologous sequence alignment and de novo prediction was used to identify the whole genome repeats. In homologous sequence alignment, sequences that were similar to known repeat sequences were identified by Repeatmasker and Repeatproteinmask (http://www.repeatmasker.org/) based on repeat sequences in the RepBase database (http://www.girinst.org/repbase/). De novo repeat sequence databases were built using tools including LTR_FINDER (https://github.com/xzhub/LTR_Finder), RepeatScout (https://github.com/mmcco/RepeatScout) and RepeatModeler (https://github.com/Dfam-consortium/RepeatModeler), with further prediction performed by Repeatmasker. Tandem repeats were extracted using TRF (http://tandem.bu.edu/trf/trf.html) by ab initio prediction.

Structural annotation of the genome incorporated ab initio prediction, homology-based prediction, and RNA-Seq-assisted prediction, which were used to annotate gene models. For homolog prediction, sequences of homologous proteins were downloaded from Ensembl and NCBI. Protein sequences were aligned to the genome using TblastN (v2.2.26; E-value ≤ 10^−5^), then the matching proteins were aligned to the homologous genome sequences for accurate spliced alignments with GeneWise (v2.4.1) software, which was used to predict the gene structure contained in each protein region. Ab initio prediction was based on Ab initio, Augustus (v3.2.3), Geneid (v1.4), Genescan (v1.0), GlimmerHMM (v3.04), and SNAP (2013-11-29). For RNA-Seq data, transcriptome read assemblies were generated with Trinity (v2.1.1) for the genome annotation. To optimize the genome annotation, the RNA-Seq reads from different tissues were aligned to genome fasta using Hisat (v2.0.4) with default parameters to identify exons region and splice positions. The alignment results were then used as input for Stringtie (v1.3.3) with default parameters for genome-based transcript assembly.

A non-redundant reference gene set was generated by merging genes predicted by ab initio prediction, homology-based prediction, and RNA-Seq-assisted prediction, as described above, with EvidenceModeler (EVM, v1.1.1) using PASA (Program to Assemble Spliced Alignment) terminal exon support, including masking transposable elements.

The gene set after gene structure annotation was aligned with known protein databases including Swiss-Prot (http://www.uniprot.org/), Nr (http://www.ncbi.nlm.nih.gov/protein), Pfam (http://pfam.xfam.org/), KEGG (http://www.genome.jp/kegg/), and InterPro (https://www.ebi.ac.uk/interpro/) to generate gene function information. Protein functions were predicted by transferring annotations from the closest BLAST hit (E-value < 10^−5^) in the Swiss-Prot20 database and DIAMOND (v0.8.22)/BLAST hit (E-value < 10^−5^) hit (E-value < 10^−5^) in the NR20 database. We also mapped to a KEGG pathway and identified the best match for each gene.

The tRNAs were predicted using the program tRNAscan-SE (http://lowelab.ucsc.edu/tRNAscan-SE/). Due to their high sequence conservation, for rRNAs, related species’ rRNA sequences were used as references to predict rRNA sequences using Blast (v2.13.0–v2.15.0). Other ncRNAs, including miRNAs and snRNAs, were identified by searching against the Rfam database with default parameters using Infernal software (http://eddylab.org/infernal/).

## 3. Results

### 3.1. Sequencing

The total output of raw Illumina DNA sequencing data was 76.1 Gb. Quality scores (Table S1, Figure S4) were mid-way between Q30 and Q40. The sequencing error rate (Figure S5) of <0.04% was much less than the median previously reported for the Illumina Novoseq 6000 [18]. The distribution of base content was essentially unchanged along the read length (Figure S6), and high-quality clean reads (Figure S7) of >98% indicated that the sequencing was of high quality (Table S2).

The total output of raw PacBio DNA sequencing data was 437 Gb. The read length distributions for polymerase reads, inserts, and subreads are shown in Table S3 and Figures S11–S13. The polymerase control N50 read length was >70 kb and the insert size N50 read length was >20 kb, which indicated that the sequencing was of high quality.

RNA was extracted from foot, mantle, digestive gland, and gill tissues. The total output of raw Illumina RNA sequencing data was 24.7 Gb. As found for Illumina DNA sequencing, quality scores (Table S4, Figures S15–S18), sequencing error (Figures S19–S22), distribution of base content along reads (Figures S23–S26), and percentage of high-quality clean reads (Figures S27–S30) indicated that the sequencing was of high quality for RNA derived from all four tissues.

### 3.2. Genome Survey and Assembly

Before assembly, genome size was estimated by kmer analysis using Kmer = 17. The K-mer distribution diagram is shown in Figure S31. The estimated genome size before and after revision, heterozygous rate, and repeat rate are shown in Table S5. The genome size estimated by the formula Genome Size = K-mer_num/Peak_depth was 1763.52 Mbp with the revised genome size 1751.71 Mbp. The heterozygous rate was 1.15%, and the repeat content 67.66%. The (K-mer = 41) assembly generated a contig N50 size of 748 bp, total contig length of 1,568,074,050 bp, scaffold N50 of 1167 bp, and total scaffold length of 1,672,366,894 bp.

PacBio reads were used for primary genome assembly together with Illumina reads for error correction [16,19]. Contigs were further assembled into scaffolds. The distributions of contig length and number with coverage depth are shown in Figures S33 and S34. The correlation of GC content (35.87%) and sequencing depth of contigs is shown in Figure S35. Total PacBio sequencing data volume (Table S6) was 437 Gb, and the coverage was 247.8× (calculated by the estimated genome size 1763.52 M from the genome survey). The final genome assembly statistics are shown in Table 1 and Figure S36. A total of 1808 contigs ranging from 10,553 bp to 5,314,528 bp resulted in a total assembled contig length representing a final genome size of 1,781,152,784 bp. GC content is shown in Table S7.

Genome completeness was evaluated by combining BUSCO and CEGMA with tools including tblastn, genewise, and geneid. *Arctica islandica* genome BUSCO assessment statistics are presented in Table S8 and Figure S37. Of 978 BUSCO groups analyzed, complete BUSCOs were at 92.7%, complete and single-copy BUSCOs at 84.2%, complete duplicated BUSCOs at 8.5%, fragmented BUSCOs at 1.6%, and missing BUSCOs at 5.7%. The percentage of complete (>70%) assembled core CEGMA genes out of 248 core genes was 84.27%, with 91.94% either completely or partially assembled (Table S9).

Illumina short reads were mapped to the assembled genome by BWA (http://bio-bwa.sourceforge.net/), and the mapping rate/coverage/depth is listed in Table S10. The read mapping rate was 97.35% with coverage of 94.45%, indicating high consistency between the assembly and the reads. The sequencing depth distribution is shown in Figure S38. For the assembled genome, 10k sliding windows were used to calculate GC contents and average sequencing depth (Figure S39) to check for possible GC bias indicative of contamination during sequencing, which indicated no contamination of the genome. Single nucleotide variation, along with tools including SAMTools, was used to order and deduplicate the BWA mapping results. Final SNP statistics are shown in Table S11. The heterozygosity SNP ratio was 0.571779% and the homozygous SNP ratio was 0.048273%, indicating high assembly accuracy.

### 3.3. Genome Annotation

**Figure 1:**
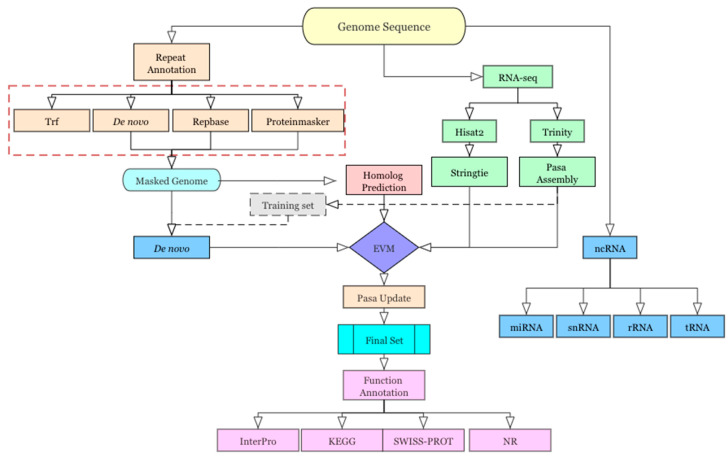
Genome annotation strategy.

Genome annotation included repeat sequence annotation, gene annotation (including gene structure annotation and gene function annotation), and non-coding RNA (ncRNA) annotation, as shown in Figure 1. A non-redundant reference gene set was generated by merging genes predicted by ab initio prediction, homology-based prediction, and RNA-Seq-assisted prediction.

Repeats found in de novo prediction were integrated with homologous repeat sequences in Repbase and annotated by RepeatMasker. The *Arctica islandica* genome included 61.59% repeat sequences (Table S12), and the annotation results are shown in Table 2. Long terminal repeats (LTR) comprised a majority of the genome. Transposable element divergence annotated by RepeatMasker based on Repbase is shown in Figure S41.

De novo gene prediction based on five tools (Table S13) showed a wide variation in gene number based on the tool used from 48,507 (Genscan) to 189,084 (GlimmerHMM). The two tools with the highest number of predicted genes also had the shortest transcript and coding sequence lengths.

Homology-based prediction (Table 3) using *Mercenaria mercenaria*, *Panopea generosa*, *Mactromeris polynyma*, *Tridacna gigas*, *Tridacna crocea*, *Serripes groenlandicus*, *Ruditapes philippinarum*, *Margaritifera margaritifera*, and *Homo sapiens* indicated the fewest (8900) genes using *Homo sapiens*, and the most (38,867) with *Panopea generosa*. Plotting the coding sequence length, exon length, exon number, gene length, and intron length (Figure S42) indicated that intron length is the most divergent across species.

All gene predictions together with transcriptome alignment were integrated to generate a non-redundant gene set (Table S14 and Figure S43) consisting of 39,509 genes with an average transcript length of 15,429 bp, an average coding sequence length of 1326 bp, and an average of six exons of 220 bp with an average intron length of 2805 bp.

**Figure 2:**
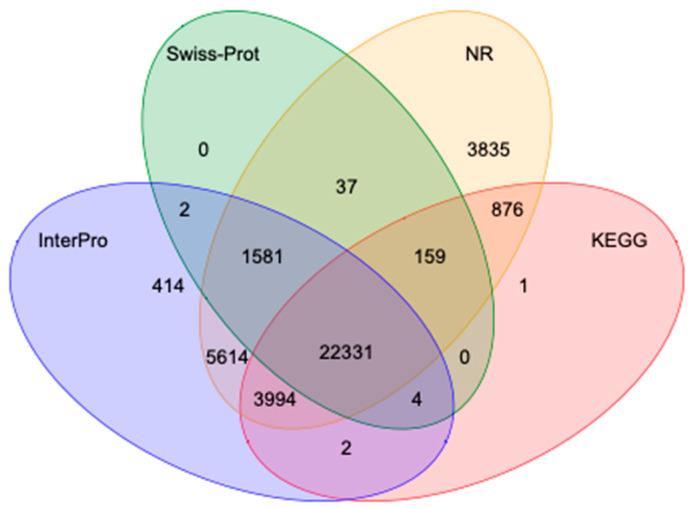
Venn diagram of predicted proteins from Swiss-Prot, non-redundant (NR), InterPro, and KEGG.

Protein sequences predicted by gene structure were aligned with known protein databases. More than 98% of the genes could be annotated across databases, with a low of 46.3% in the Gene Ontology database and 97.3% in the non-redundant database (Table S15). The overlap of the protein databases is shown in Figure 2.

Annotation of non-coding RNAs included tRNAs that were predicted by structural features, rRNAs by homology with the species described above, and miRNAs and snRNAs predicted based on covariance models. Predicted non-coding RNAs in the *Arctica islandica* genome (Table 4) include 801 miRNAs, 11,114 tRNAs, 909 rRNAs, and 349 snRNAs.

## 4. Discussion

*Arctica islandica* (ocean quahog, Linnaeus, 1767), the longest-lived non-colonial animal, is a member of the order Veneroida, family Arcticidae [20], which has a range that spans the continental shelves of the west coast of Europe and east coast of North America. These animals can be found buried in the sediments of muddy sands and, as a suspension feeder, pump ocean water through their muscular siphons [21]. The name mahogany clam derives from a golden brown to mahogany pigmentation deposited in the shell that may darken to black due to iron deposits [22]. *Arctica islandica* is commercially harvested in North America, but it is its extreme longevity and body plan that includes heart, gills, digestive system, kidney, muscular system, neurons, and mineralized shell [23] that position it as an important animal model for aging research.

Despite this potential scientific value, only a limited number of genetic studies have previously been published on *Arctica islandica*. Transcriptomes from clams exposed to different oxygen conditions have been generated to study oxidative stress genes [24]. Analysis of telomeres has been reported [25], the mitochondrial genome has been sequenced [26], and cytogenetic analysis has been conducted that included a reference to a genome sequencing project in progress [27].

We used long-read and short-read DNA sequencing and RNA sequencing from four tissues to de novo assemble the genome of *Arctica islandica*. Long-read sequencing has enabled the assembly of genomes de novo with much greater accuracy and completeness than short reads. We used Pacific Biosciences (PacBio) High-Fidelity (HiFi) reads of 10–20 kb in length that have an error rate below 0.5% as the backbone data that have been used to generate high-quality assemblies [28]. Illumina short reads were then used for polishing. Despite multiple attempts (Supplementary Material Method S5.9 and Figure S40), we were unable to generate Hi-C sequencing data of sufficient quality to allow for a genome assembly at the chromosomal level. The DNA input for Hi-C involves fixation of cells in situ, which can have tissue-specific effects. Hi-C sequencing has been used to assemble other clam genomes [29] at the chromosomal level [30], including at least one with an identical number of chromosomes [27].

The primary genome assembly was at a high coverage of 248.7×, with sequencing statistics indicating high quality. Complete BUSCOs were at 92.7% and the short-read mapping rate onto the assembly was >97% with 94% coverage. The genome size of 1.75 GB is similar to other clam genomes (Table 5), including the 1.78 GB *Mercenaria mercenaria* hard clam genome [31], 1.47 GB *Panopea generosa* Pacific geoduck [8], 1.41 Gb Manila clam *Ruditapes philippinarum* [32], 1.30 Gb Crocus Giant Clam *Tridacna crocea* [12], 1.18 GB giant clam *Tridacna gigas* [10], 0.978 GB purple butter clam *Saxidomus purpuratus* [33], 2.4 Gb European freshwater pearl mussel *Margaritifera margaritifera* [34], 0.793 GB Arctic surf clam *Mactromeris polynyma* [35], and 1.8 GB Greenland cockle *Serripes groenlandicus* [35].

The results of repeat annotation showed that repeats accounted for 61.59% in the *Arctica islandica* genome, a greater percentage than the 49.11% of the *Mercenaria mercenaria* hard clam genome [31], 57.99% of the *Panopea generosa* Pacific geoduck [8], 39.7% of the Manila clam *Ruditapes philippinarum* [32], 55.83% of the Crocus Giant Clam *Tridacna crocea* [12], 50.81% of the purple butter clam *Saxidomus purpuratus* [33], and 57.32% of the genome of the European freshwater pearl mussel *Margaritifera margaritifera* [34] (Table 5).

The non-redundant gene set consisted of 39,509 genes, with more than 98% that could be annotated. The predicted number of genes in other clam genomes (Table 5) included 34,283 genes in the *Mercenaria mercenaria* hard clam genome [31], 35,034 genes in the *Panopea generosa* Pacific geoduck genome [8], 34,505 genes in the Manila clam *Ruditapes philippinarum* genome [32], 25,440 genes in the Crocus Giant Clam *Tridacna crocea* genome [12], 37,598 genes in the giant clam *Tridacna gigas* genome [10], 37,690 genes in the purple butter clam *Saxidomus purpuratus* genome [33], and 35,119 genes in the European freshwater pearl mussel *Margaritifera margaritifera* genome [34]. Other than the Crocus Giant Clam *Tridacna crocea*, the predicted number of genes is surprisingly similar across species despite significant differences in gene prediction pipelines used in the genome assemblies.

The reported maximum longevities of these other bivalve species include 106 years for the *Mercenaria mercenaria* hard clam [13,36], 168 years for the *Panopea generosa* Pacific geoduck [13], 7 years for the Manila clam *Ruditapes philippinarum* [37], >100 years for the giant clam *Tridacna gigas* [5], 100 years for the crocus giant clam *Tridacna crocea* [38], 23 years for the purple butter clam *Saxidomus purpuratus* [39], 190 years for the European freshwater pearl mussel *Margaritifera margaritifera* [40], 60 years for the Arctic surf clam *Mactromeris polynyma* [13], and 48 years for the Greenland cockle *Serripes groenlandicus* [14]. Only about forty of all known animal species, including humans, have documented maximum life spans greater than 100 years, i.e., are centenarian species [41]. Comparative genomic analysis among long-lived and short-lived species can be a powerful approach to investigating aging [42], as is the analysis of the evolution of extreme longevity [43,44], which enables an unbiased approach to the discovery of known and/or new genes and pathways that regulate longevity. However, most gerontological studies to date have focused primarily on a relatively low number of model systems and/or conducted analyses largely through the lens of known hallmarks [45]. There has also been an emphasis directed toward research on health span, rather than maximum life span, both of which have potential medical and scientific importance [46]. We suggest that bivalve clams, which represent a diverse class of Mollusca, may be ideal for determining the genetic determinants of extreme longevity using evolutionary approaches.

## 5. Conclusions

We have generated the first high-quality assembly with extensive annotation of the Arctica islandica genome using short and long read sequencing. As the longest-lived non-colonial animal species, these data will contribute to the comparative and evolutionary analyses of the genomic basis of extreme longevity. 

## Figures and Tables

**Table 1:** Final genome assembly statistics.

Assembly Metric	Result
Total assembled contig length (bp)	1,781,152,784
Total number of contigs	1808
Maximum contig length (bp)	5,314,528
Minimum contig length (bp)	10,553
N50 contig length (bp)	1,470,266
N50 contig number (#)	390

**Table 2:** Repeat composition of the *Arctica islandica* genome.

	Denovo + Repbase *		TE Proteins **		Combined TEs ***	
	Length (bp)	% in Genome	Length (bp)	% in Genome	Length (bp)	% in Genome
**DNA**	23,116,728	1.30	8,479,226	0.48	30,079,574	1.69
**LINE**	15,346,565	0.86	43,780,071	2.46	53,675,585	3.01
**SINE**	6472	0.00	0	0	6472	0.00
**LTR**	938,560,330	52.69	10,599,309	0.60	940,678,889	52.81
**Unknown**	66,824,036	3.75	0	0	66,824,036	3.75
**TOTAL**	1,029,426,374	57.80	62,848,757	3.53	1,046,221,933	58.74

* Denovo + Repbase refers to transposable elements that are predicted by RepeatModeler, RepeatScout, Piler, and LTR_FINDER combined with RepBase, and are integrated by Uclust in an 80-80-80 principle and are annotated by RepeatMasker. ** TE proteins refer to transposable elements that are predicted based on RepBase protein data and are annotated by RepeatProteinMask. *** Combined TEs are non-redundant transposable elements of the results above. Unknown refers to repeats that could not be classified by RepeatMasker. TOTAL is the non-redundant results of all the above.

**Table 3:** Homologous protein-coding gene prediction.

Gene Set	Number	Average Transcript Length (bp)	Average CDS Length (bp)	Average Exons per Gene	Average Exon Length (bp)	Average Intron Length (bp)
*Homo sapiens*	8900	9311	998	4	227	2442
*Tridacna crocea*	29,771	9598	1054	5	227	2343
*Mactromeris polynyma*	23,513	11,181	1123	5	229	2581
*Panopea generosa*	38,867	6564	866	3	252	2346
*Margaritifera margaritifera*	30,513	7111	882	4	242	2352
*Mercenaria mercenaria*	35,714	10,789	1145	5	231	2440
*Serripes groenlandicus*	22,925	10,114	1084	5	235	2503
*Tridacna gigas*	27,166	10,232	1074	5	224	2408
*Ruditapes philippinarum*	34,805	10,770	1162	5	233	2414

**Table 4:** Predicted non-coding RNA (rRNA, tRNA, snRNA, miRNA) in the *Arctica islandica* genome.

Type of ncRNA	Copy Number	Average Length (bp)	Total Length (bp)	% of Genome
**miRNA**	801	118.50	94,919	0.005329
**tRNA**	11,114	73.00	811,284	0.045548
**rRNA**	909	214.65	195,121	0.010955
18S	793	231.59	183,653	0.010311
28S	28	119.46	3345	0.000188
5.8S	0	0	0	0
5S	88	92.31	8123	0.000456
**snRNA**	349	140.70	49,106	0.002757
CD-box	98	108.98	10,680	0.000600
HACA-box	36	191.56	6896	0.000387
splicing	198	141.93	28,102	0.001578
scaRNA	8	191.38	1531	0.000086
unknown	9	210.78	1897	0.000107

**Table 5:** Genome size, repeat percentage, gene number, and maximum life spans (MLS) of species in the class Mollusca, order Bivalvia.

Common Name	Genus	Species	Order	Genome Size (GB)	Repeat Percentage	Number of Genes	MLS (Years)
Ocean Quahog	*Arctica*	*islandica*	Venerida	1.75	61.59	39,509	>500
European Freshwater Pearl Mussel	*Margaritifera*	*margaritifera*	Unionida	2.4	57.32	35,119	190
Pacific Geoduck	*Panopea*	*generosa*	Adapedonta	1.47	57.99	35,034	168
Hard Clam	*Mercenaria*	*mercenaria*	Venerida	1.78	49.11	34,283	106
Giant Clam	*Tridacna*	*gigas*	Cardiida	1.18	-	37,598	>100
Crocus Giant Clam	*Tridacna*	*crocea*	Cardiida	1.30	55.83	25,440	100
Arctic Surf Clam	*Mactromeris*	*polynyma*	Venerida	0.793	-	-	60
Greenland Cockle	*Serripes*	*groenlandicus*	Cardiida	1.8	-	-	48
Purple Butter Clam	*Saxidomus*	*purpuratus*	Venerida	0.978	50.81	37,690	23
Manila Clam	*Ruditapes*	*philippinarum*	Venerida	1.41	39.7	34,505	7

## Data Availability

The data presented in this study are available on request from the corresponding author. Data will be provided for requests with no commercial interests.

## Supplementary Materials

The following supporting information can be downloaded at: https://www.mdpi.com/article/10.3390/ani15050690/s1, Figure S1: Image of live *Arctica islandica*; Method S1: Short read Illumina DNA Sequencing; Figure S2: Electrophoresis of DNA used for sequencing; Figure S3: Workflow of Illumina sequencing; Figure S4: Distribution of Illumina sequencing quality scores; Figure S5: Illumina error rate by read base position; Figure S6: A/T/G/C distribution by read base position in Illumina reads; Figure S7: Illumina raw read filtering results; Table S1: Sequencing error rate (e) and sequencing base quality value (Qphred); Table S2: DNA Sequence Data Quality Summary; Method S2: Long Read PacBio DNA Sequencing; Figure S8: PacBio sequencing workflow; Figure S9: Distribution of DNA size after fragmentation; Figure S10: Consensus read process; Figure S11: Polymerase length distribution; Figure S12: Insert size length distribution; Figure S13: Subreads length distribution; Table S3: PacBio sequence data metrics; Method S3: Short read Illumina RNA Sequencing; Figure S14: Illumina RNA Library Construction, Quality Control and Sequencing.; Figure S15: Quality score distribution of Illumina RNA sequencing reads (m245-Foot RNA); Figure S16: Quality score distribution of Illumina RNA sequencing reads (m236 Mantle RNA); Figure S17: Quality score distribution of Illumina RNA sequencing reads (m229 Digestive Gland RNA); Figure S18: Quality score distribution of Illumina RNA sequencing reads (m227 Gill RNA); Figure S19: Illumina RNA sequencing error rate by read base position (m245-Foot RNA); Figure S20: Illumina RNA sequencing error rate by read base position (m236 Mantle RNA); Figure S21: Illumina RNA sequencing error rate by read base position (m229 Digestive Gland RNA): Figure S22: Illumina RNA sequencing error rate by read base position (m227 Gill RNA); Figure S23: Illumina RNA sequencing A/T/G/C distribution by read base position (m245-Foot RNA); Figure S24: Illumina RNA sequencing A/T/G/C distribution by read base position (m236 Mantle RNA); Figure S25: Illumina RNA sequencing A/T/G/C distribution by read base position (m229 Digestive Gland RNA); Figure S26: Illumina RNA sequencing A/T/G/C distribution by read base position (m227 Gill RNA; Figure S27: Illumina RNA raw read filtering results (m245-Foot RNA); Figure S28: Illumina RNA raw read filtering results (m236 Mantle RNA); Figure S29: Illumina RNA raw read filtering results (m229 Digestive Gland RNA); Figure S30: Illumina RNA raw read filtering results (m227 Gill RNA); Table S4: RNA Sequencing Data Quality Summary; Method S4: Genome Survey; Figure S31: K-mer distribution; Table S5: Kmer analysis summary; Method S5: Primary Genome Assembly and Assessment; Figure S32: Genome assembly strategy; Figure S33: Distribution of the contig length and coverage depth; Figure S34; Distribution of the contig numbers and coverage depth; Figure S35: Correlation of GC content and sequencing depth of contigs; Figure S36: Cumulative contig summary (shortest to longest); Figure S37: BUSCO assessment statistics; Figure S38: Sequencing depth distribution; Figure S39: Sequencing depth distribution versus GC content; Figure S40: Bioanalyzer results for Hi-C libraries prepared from 4 tissues; Table S6: Sequence coverage; Table S7: Genome A/T/G/C contents; Table S8: BUSCO assessment statistics; Table S9: CEGMA assessment statistics; Table S10: *Arctica* genome read mapping rate/coverage/depth statistics; Table S11: SNP statistics; Method S6: Genome Annotation; Figure S41: TE divergence annotated by RepeatMasker based on Repbase; Figure S42: Gene structure length species comparison line chart; Figure S43: Venn diagram of gene set evidence support; Table S12: Repeat size and percentage of genome; Table S13: De novo gene prediction based on 5 tools; Table S14: Final non-redundant gene set; Table S15: Proteins predicted by *Arctica islandica* gene structure aligned with known proteins in various databases; Method S7: Versions of Genome Assembly Tools.
