# Supplementary material for: Genome Assembly of Arctica islandica, the Longest-Lived Non-Colonial Animal Species
Source: Animals (Basel). 2025 Feb 27;15(5):690. doi: 10.3390/ani15050690 (PMC11899663; doi:10.3390/ani15050690)
Supplement: Supplementary file 1 [file animals-15-00690-s001.zip › animals-3355323-supplementary.pdf]

Supplementary Material Appendix A

Genome assembly of *Arctica islandica*, the longest lived non-colonial animal species.

Glenn S. Gerhard[1*], John Allard[2,3], Scott Kaniper[1], Dorret Lynch[1], Hayan Lee[4], and Sudhir Kumar[2,3]

1 Lewis Katz School of Medicine at Temple University, Philadelphia, PA 19140, USA

2 Institute for Genomics and Evolutionary Medicine, Temple University, Philadelphia, PA 19122, USA

3 Department of Biology, Temple University, Philadelphia, PA 19122, USA

4 Cancer Epigenetics Institute, Fox Chase Cancer Cancer, Philadelphia, PA 19111, USA

**Corresponding author* (gsgerhard@temple.edu)

Short Title: *Arctica islandica* genome

Supplementary Figures

S1. Image of live *Arctica islandica*.

Supplementary Methods

Method S1. Short read Illumina DNA Sequencing

Figure S2. Electrophoresis of DNA used for sequencing.

Figure S3. Workflow of Illumina sequencing.

Figure S4. Distribution of Illumina sequencing quality scores.

Figure S5. Illumina error rate by read base position.

Figure S6. A/T/G/C distribution by read base position in Illumina reads.

Figure S7. Illumina raw read filtering results.

Table S1. Sequencing error rate (e) and sequencing base quality value (Q_{phred}).

Table S2. DNA Sequence Data Quality Summary

Method S2. Long Read PacBio DNA Sequencing

Figure S8. PacBio sequencing workflow.

Figure S9. Distribution of DNA size after fragmentation

Figure S10. Consensus read process.

Figure S11. Polymerase length distribution.

Figure S12. Insert size length distribution.

Figure S13. Subreads length distribution.

Table S3. PacBio sequence data metrics

Method S3. Short read Illumina RNA Sequencing

Figure S14. Illumina RNA Library Construction, Quality Control and Sequencing.

Figure S15. Quality score distribution of Illumina RNA sequencing reads (m245-Foot RNA).

Figure S16. Quality score distribution of Illumina RNA sequencing reads (m236 Mantle RNA).

Figure S17. Quality score distribution of Illumina RNA sequencing reads (m229 Digestive Gland RNA).

Figure S18. Quality score distribution of Illumina RNA sequencing reads (m227 Gill RNA).

Figure S19. Illumina RNA sequencing error rate by read base position (m245-Foot RNA).

Figure S20. Illumina RNA sequencing error rate by read base position (m236 Mantle RNA).

Figure S21. Illumina RNA sequencing error rate by read base position (m229 Digestive Gland RNA).

Figure S22. Illumina RNA sequencing error rate by read base position (m227 Gill RNA).

Figure S23. Illumina RNA sequencing A/T/G/C distribution by read base position (m245-Foot RNA).

Figure S24. Illumina RNA sequencing A/T/G/C distribution by read base position (m236 Mantle RNA).

Figure S25. Illumina RNA sequencing A/T/G/C distribution by read base position (m229 Digestive Gland RNA).

Figure S26. Illumina RNA sequencing A/T/G/C distribution by read base position (m227 Gill RNA).

Figure S27. Illumina RNA raw read filtering results (m245-Foot RNA).

Figure S28. Illumina RNA raw read filtering results (m236 Mantle RNA).

Figure S29. Illumina RNA raw read filtering results (m229 Digestive Gland RNA).

Figure S30. Illumina RNA raw read filtering results (m227 Gill RNA).

Table S4. RNA Sequencing Data Quality Summary

Method S4. Genome Survey

Figure S31. K-mer distribution.

Table S5. Kmer analysis summary.

Method S5. Primary Genome Assembly and Assessment

Figure S32. Genome assembly strategy.

Figure S33. Distribution of the contig length and coverage depth.

Figure S34. Distribution of the contig numbers and coverage depth.

Figure S35. Correlation of GC content and sequencing depth of contigs.

Figure S36. Cumulative contig summary (shortest to longest).

Figure S37. BUSCO assessment statistics.

Figure S38. Sequencing depth distribution.

Figure S39. Sequencing depth distribution versus GC content.

Figure S40. Bioanalyzer results for Hi-C libraries prepared from 4 tissues.

Table S6. Sequence coverage.

Table S7. Genome A/T/G/C contents.

Table S8. BUSCO assessment statistics.

Table S9. CEGMA assessment statistics.

Table S10. Arctica genome read mapping rate/coverage/depth statistics.

Table S11. SNP statistics.

Method S6. Genome Annotation

Figure S41. TE divergence annotated by RepeatMasker based on Repbase.

Figure S42. Gene structure length species comparison line chart.

Figure S43. Venn diagram of gene set evidence support.

Table S12. Repeat size and percentage of genome.

Table S13. De novo gene prediction based on 5 tools.

Table S14. Final non-redundant gene set.

Table S15. Proteins predicted by Arctica islandica gene structure aligned with known proteins in various databases.

Method S7. Versions of Genome Assembly Tools

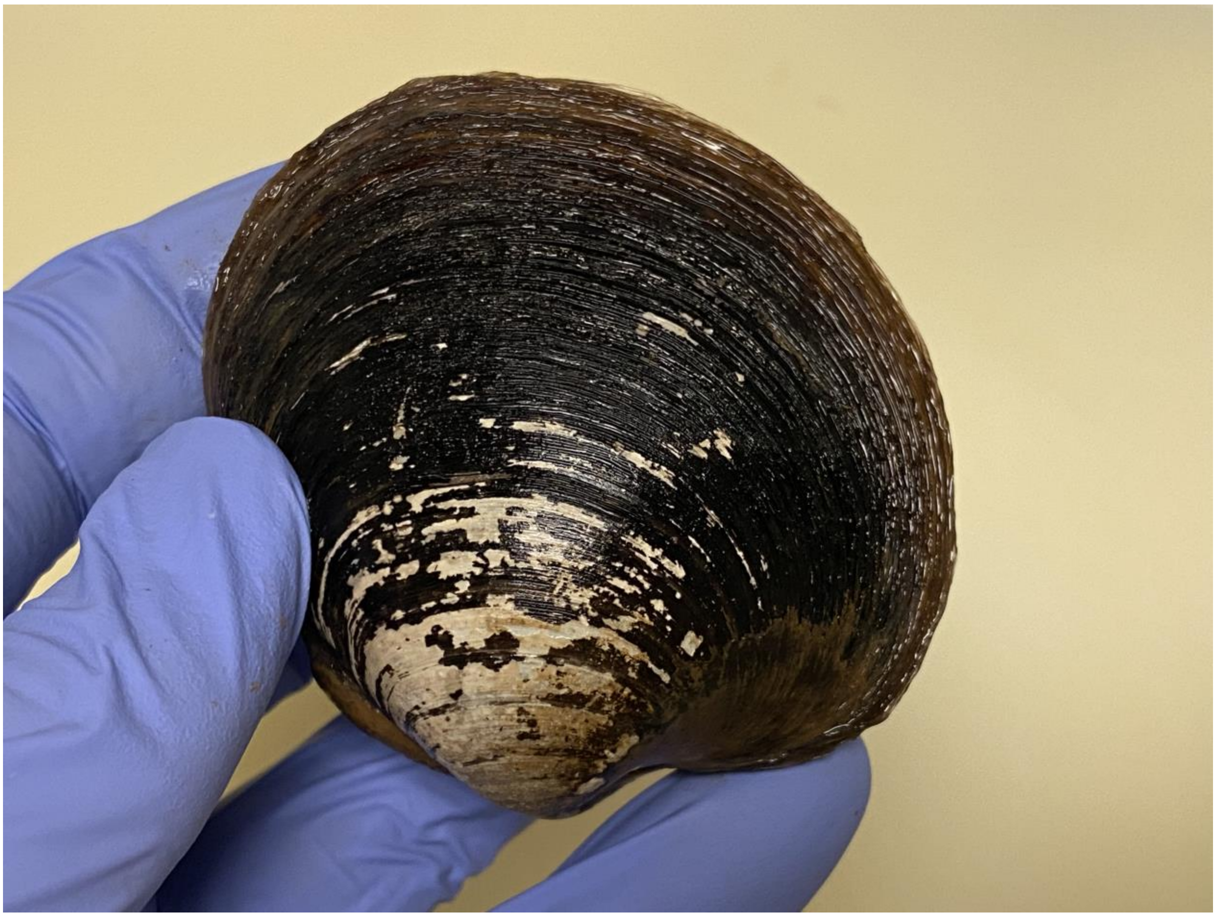

Figure S1. Image of live *Arctica islandica*.

Method S1. Short read Illumina DNA Sequencing

Method S1.1. Library Construction, Quality Control and Sequencing

Total DNA was extracted from 300 mg of gill tissue using the Qiagen DNeasy Blood & Tissue Kit, quantified using Nanodrop D-1000 and stored at -80°C. The DNA concentration was 598 ng/ul, A260/280 was 1.86, and the A260/230 was 1.75. Agarose gel electrophoresis (1% run at 180 volts for 20 minutes) assessment of quality indicated high molecular weight without significant degradation (Figure S2A). Pulsed field gel electrophoresis PFGE (0.8%; 5-80K wave-form type run for 17h) also showed high molecular weight DNA without significant degradation (Figure S2B).

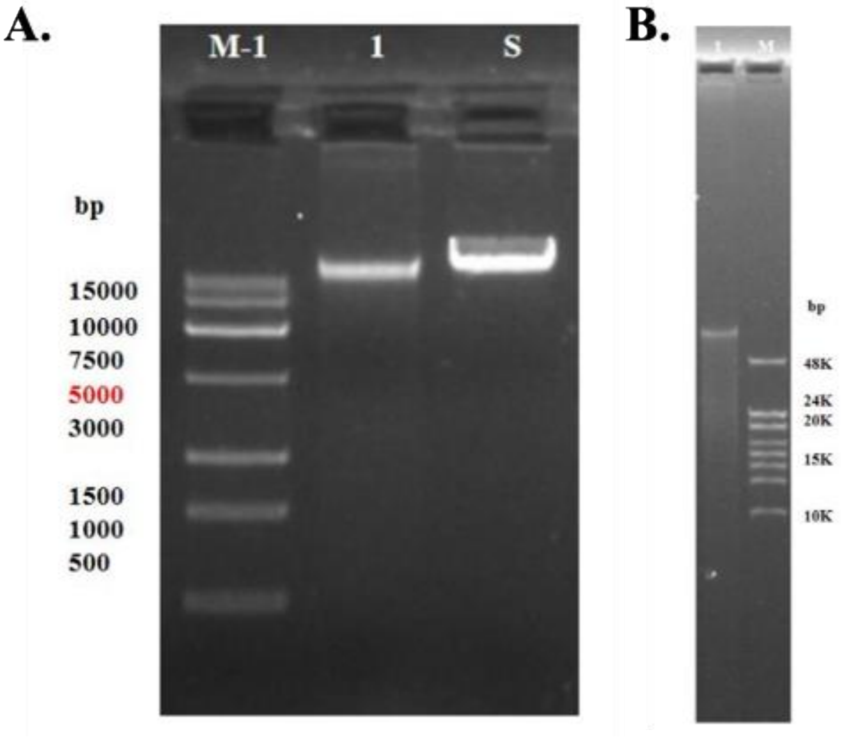

Figure S2. Electrophoresis of DNA used for sequencing. A. Agarose gel electrophoresis. B. Pulsed field gel electrophoresis.

Genomic DNA was fragmented using a Megaruptor (Diagenode, Liege, Belgium) using a proprietary algorithm (Novogene). The sheared DNA fragments were used to prepare pair-end libraries using the Rapid Plus DNA Lib Prep Kit according to the manufacturer's instructions (Illumina Inc., San Diego, CA) with a visually verified average insert size meeting quality control range of 320-350 bp confirmed via Bioanalyzer (Agilent, Santa Clara, CA) analysis. The obtained fragments were end repaired, A-tailed and further ligated with Illumina adapters. The fragments with adapters were PCR amplified, size selected, and purified. The workflow of library construction is shown in Figure S3.

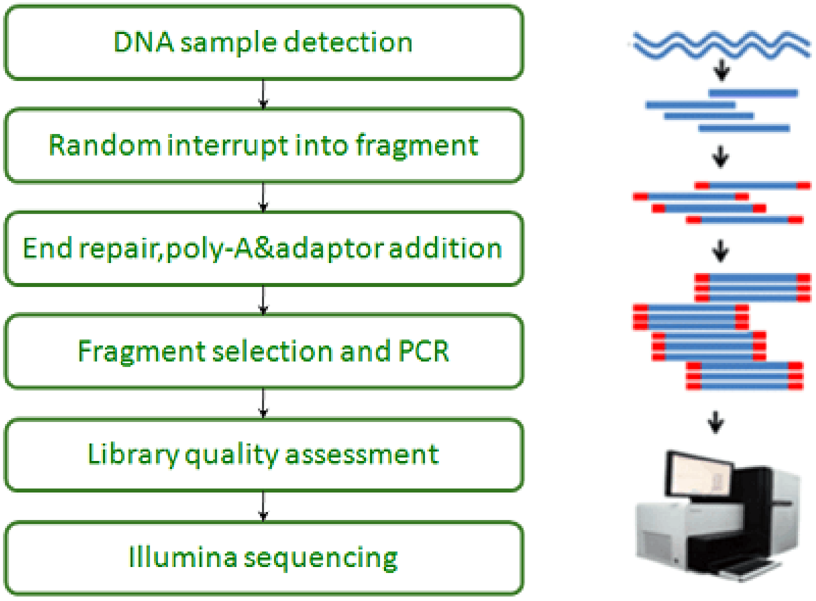

Figure S3. Workflow of Illumina sequencing.

Quantified (Qubit and PCR) libraries were pooled and sequenced in two independent lanes of a Novoseq 6000 (Illumina, Inc.) using 150-bp pair-end format. The raw data were converted to single-sample FASTQ files through base calling, and after filtering interference

information such as adaptors and low-quality reads, the clean data FASTQ files for each sample were used for further bioinformatics analyses.

Method S1.2. Distribution of Sequencing Quality

To determine sequencing quality, Q-scores were used. “Error/base” ("e") represents the sequence error rate, with $Q_{phred}=-10\log_{10}(e)$ representing the base quality value. The relationship between sequencing error rate (Error/base) and sequencing base quality value (Q_{phred}) is shown in the Table S1 below.

Table S1. Sequencing error rate and sequencing base quality value (Q_{phred}).

**Phred score**	**Error/base**	**Correct base**	**Q-score**
10	1/10	90%	Q10
20	1/100	99%	Q20
30	1/1000	99.9%	Q30
40	1/10000	99.99%	Q40

The distribution of quality scores is shown in Figure S4 where the base position along reads is on the horizontal axis and the quality score is on the vertical axis.

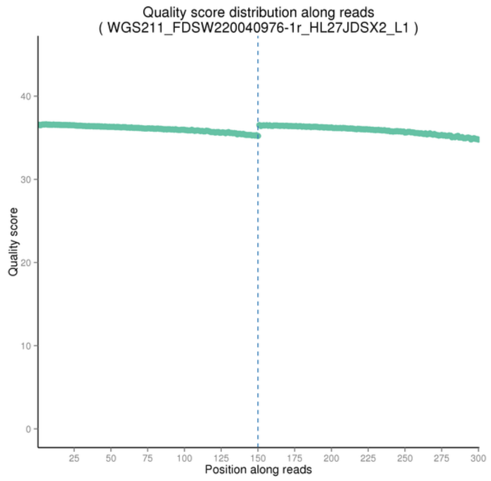

Figure S4. Distribution of Illumina sequencing quality scores.

Method S1.3. Sequencing Error

For Illumina SBS technology, the distribution of sequencing error rate has two features:

(1) Error rate increases along the length of sequenced reads because of the consumption of sequencing reagents.

(2) A high error rate occurs in the first six bases because the random hex-primers and RNA templates bind incompletely in the process of cDNA synthesis.

The error rate is shown in Figure S5**.** The base position along the reads is on the horizontal axis and the single base error rate is on the vertical axis.

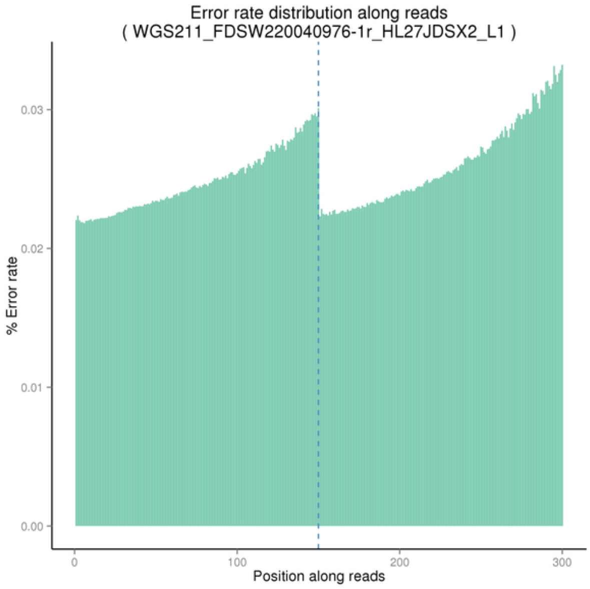

Figure S5. Illumina error rate by read base position.

Method S1.4. Distribution of A/T/G/C Bases.

The content of AT and GC should be equal at each sequencing cycle and be constant and stable throughout the whole sequencing procedure. In practice, the first 6 to 7 nucleotides will fluctuate due to the primer amplification bias.

The distribution of GC content is shown in Figure S6**.** The base position along reads is on the horizontal axis, and the percentage of each base is on the vertical axis.

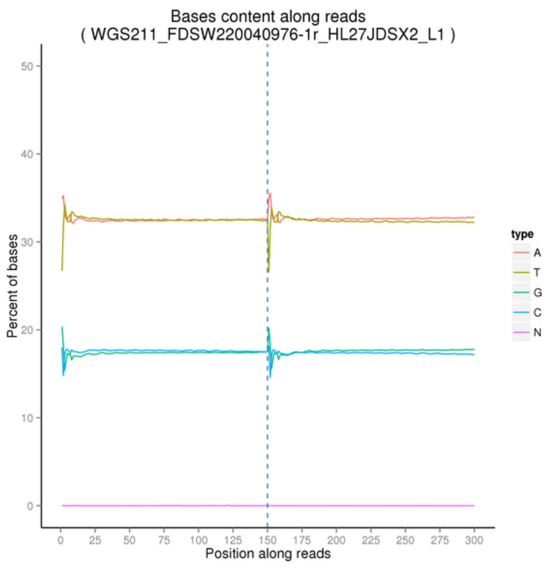

Figure S6. A/T/G/C distribution by read base position in Illumina reads.

Method S1.5. Raw Data Filtering

The sequenced reads (raw reads) were filtered to exclude low quality reads and adapters as follows:

(1) Remove reads containing adapters.

(2) Remove reads containing N > 10% (N represents a base that cannot be determined).

(3) Remove reads containing low quality (Qscore<= 5) bases.

Sequences of adapters

5' Adapter:

5'-AGATCGGAAGAGCGTCGTGTAGGGAAAGAGTGTAGATCTCGGTGGTCGCCGTATCATT-3'

3' Adapter:

5'-GATCGGAAGAGCACACGTCTGAACTCCAGTCACGGATGACTATCTCGTATGCCGTCTTCTGCTTG-3'

Raw read filtering results are shown in Figure S7 based on:

(1) Adapter related: (reads containing adapter) / (total raw reads)

(2) Containing N: (reads with more than 10% N) / (total raw reads)

(3) Low quality: (reads of low quality) / (total raw reads)

(4) Clean reads: (clean reads) / (total raw reads)

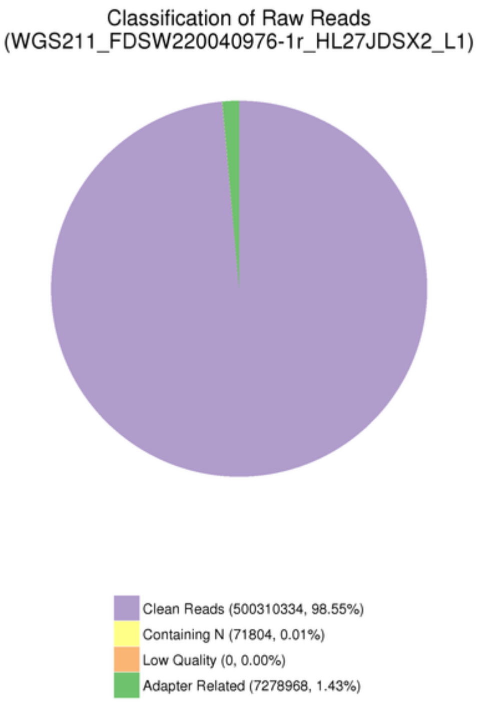

Figure S7. Illumina raw read filtering results.

Method S1.6 Summary of Sequencing Data

The total output of data from the sequencer: Raw data 76.1 Gb.

The detail statistics for the quality of sequencing data are shown in Table S2**.**

Table S2. DNA Sequence Data Quality Summary

**Raw reads**	**Raw data (Gb)**	**Effective (%)**	**Error (%)**	**Q20 (%)**	**Q30 (%)**	**GC (%)**
507661106	76.1	98.55	0.03	97.82	93.78	34.96

Raw data: (Raw reads) * (sequence length), calculated in Gigabases.

Effective: (Clean reads/Raw reads)*100%

Error: base error rate

Q20, Q30: (Base count of Phred value > 20 or 30) / (Total base count)

GC: (G & C base count) / (Total base count)

Method S2. Long Read PacBio DNA Sequencing

Method S2.1. Sequencing workflow

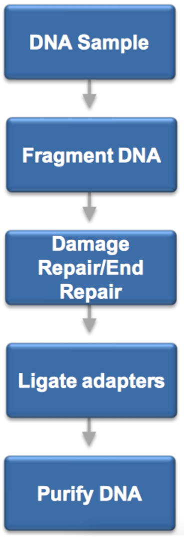

Figure S8. PacBio sequencing workflow.

The DNA used for Illumina sequencing was also used for PacBio sequencing. The SMRTbell™ Express Template Prep Kit 2.0 following PacBio library instructions as shown in Figure S8 was used to construct libraries. After QC, genomic DNA was fragmented using a proprietary (Novogene) protocol on a Megaruptor (Covaris, Woburn, MA) with smear analysis (Figure S9) showing a fragment size range from 2,747 bp to 254,361 bp with an average size of 29,784 bp and a peak of the distribution at 24,363 bp. After purification by magnetic beads damage ends were repaired. After A-tailing, DNA fragments are ligated to the hairpin adaptors, and the reaction in heat-killed. Damaged or non-intact SMRTbell templates are removed by nuclease treatment, and remaining products are purified by magnet beads. After size-selection, SMRTbell libraries were purified by magnetic beads and sequenced in ZMWs of SMRT® Cells by a PacBio Sequel II using the standard protocol of the PacBio Sequel II binding kit 2.0 and PacBio Sequel II sequencing kit 2.0.

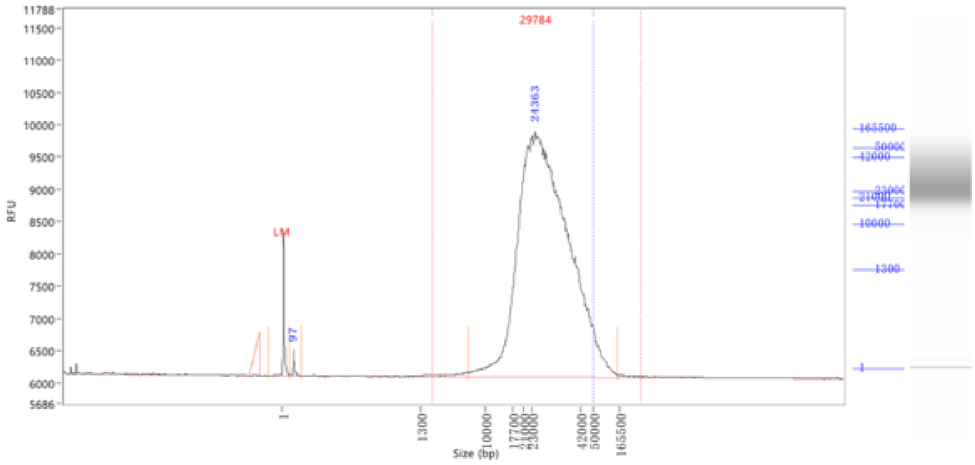

Figure S9. Distribution of DNA size after fragmentation.

Method S2.2 Data QC

The consensus read generation process is shown in Figure S10.

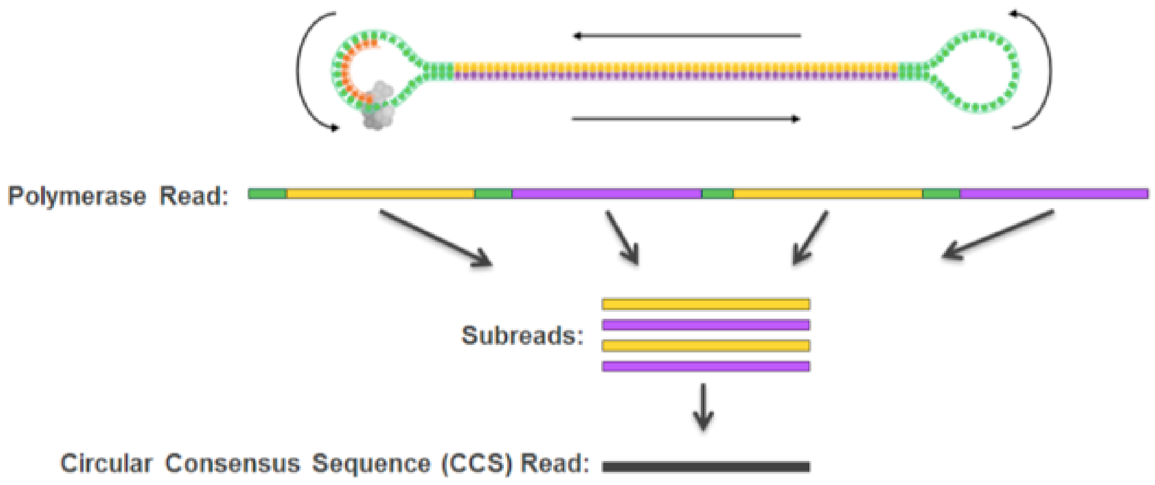

Figure S10. Consensus read process.

Original reads of circular SMRTbell templates include two adaptors called polymerase reads. SAMPLE.subreads.bam files are slightly smaller than the original data due to adaptor removal.

Insert length is estimated by the length of subreads from the same polymerase reads. After adaptor trimming of SAMPLE.subreads.bam, subreads are generated and filtered by minimum length = 50 and used for downstream analysis.

Table S3. PacBio sequence data metrics.

Read_type	Read_base	Read_Number	Read_length(max)	Read_length(mean)	Read_length(N50)
Polymerase	438,462,723,664	13,825,430	478,032	31,714	84,830
Insertsize	218,520,931,295	13,825,430	478,032	15,805	23,362
Subreads	437,554,729,300	31,362,897	478,032	13,951	21,489

The read length statistics and distributions for polymerase reads, inserts, and subreads are shown in Table S3 and Figures S11, S12, and S13. Polymerase reads include sequences of nucleotides incorporated by the DNA polymerase while reading the circular SMRTbell template. They can also include sequences from adapters as well as inserts. Polymerase read metrics primarily reflect run parameters rather than insert size distribution. Polymerase reads are trimmed to include only the high-quality regions. Sample quality is a major factor in polymerase read metrics. Each polymerase read is partitioned to form one or more subreads containing sequences from a single pass of a polymerase on a single strand of an insert within a SMRTbell template and noadapter sequences. The subreads contain the full set of quality values and kinetic measurements.

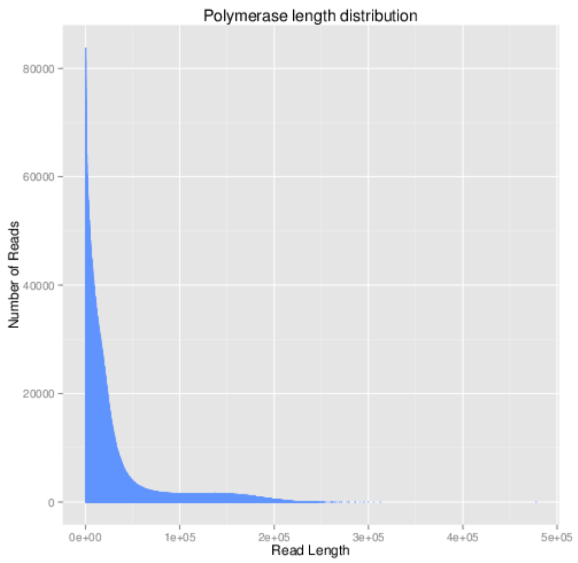

Figure S11. Polymerase length distribution.

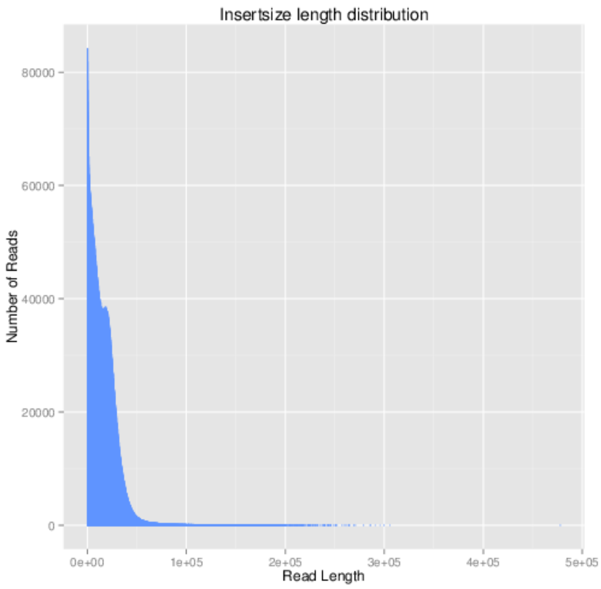

Figure S12. Insert size length distribution.

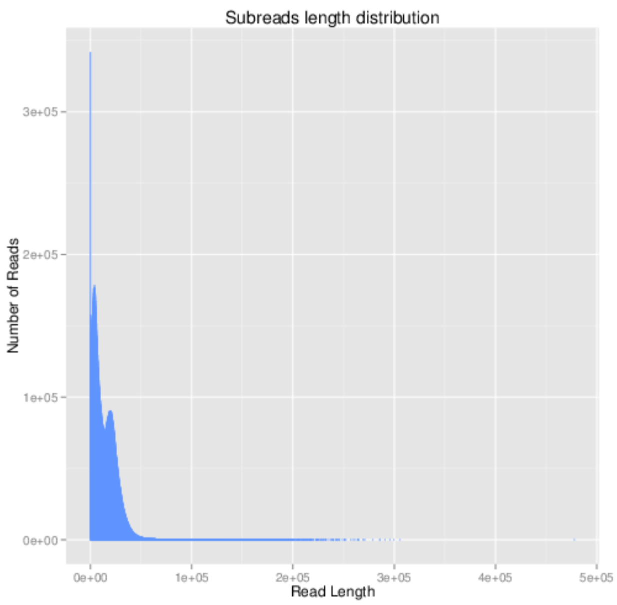

Figure S13. Subreads length distribution.

Method S3. Short read Illumina RNA Sequencing

Method S3.1. Library Construction, Quality Control and Sequencing

Total RNA was extracted from the foot, mantle, digestive gland, and gill tissues using the QiaQuick RNA kit (Qiagen). Messenger RNA was purified from total RNA using poly-T oligo-attached magnetic beads. After fragmentation using a proprietary (Novogene) protocol on a Megaruptor (Covaris, Woburn, MA), the first strand cDNA was synthesized using random hexamer primers, followed by the second strand cDNA synthesis. The library (Fast RNA-seq Lib Prep Kit V2) was ready after end repair, A-tailing, adapter ligation, size selection, amplification, and purification as shown in Figure S14. The library was checked with Qubit and real-time PCR was used for quantification and bioanalyzer for size distribution. Quantified libraries were pooled and sequenced on a Novoseq 6000 (Illumina).

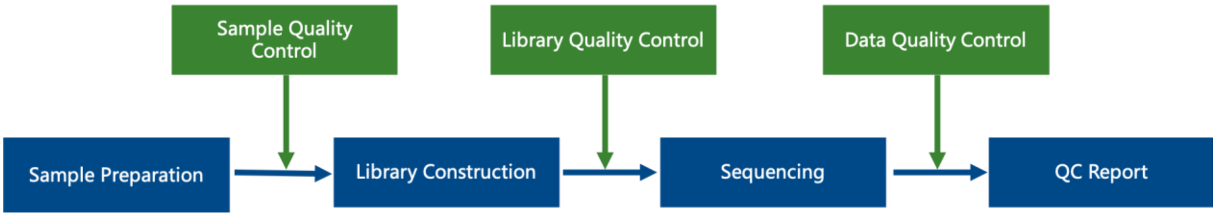

Figure S14. Illumina RNA Library Construction, Quality Control and Sequencing.

Method S3.2. Distribution of Illumina RNA sequencing quality

Sequencing quality was determined as described above in Method S1.2 and Table S1. The distribution of quality scores is shown in Figures S15 (m245-Foot RNA), S16 (m236 Mantle RNA), S17 (m229 Digestive Gland RNA), and S18 (m227 Gill RNA), where the base position along reads is on the horizontal axis and the quality score is on the vertical axis.

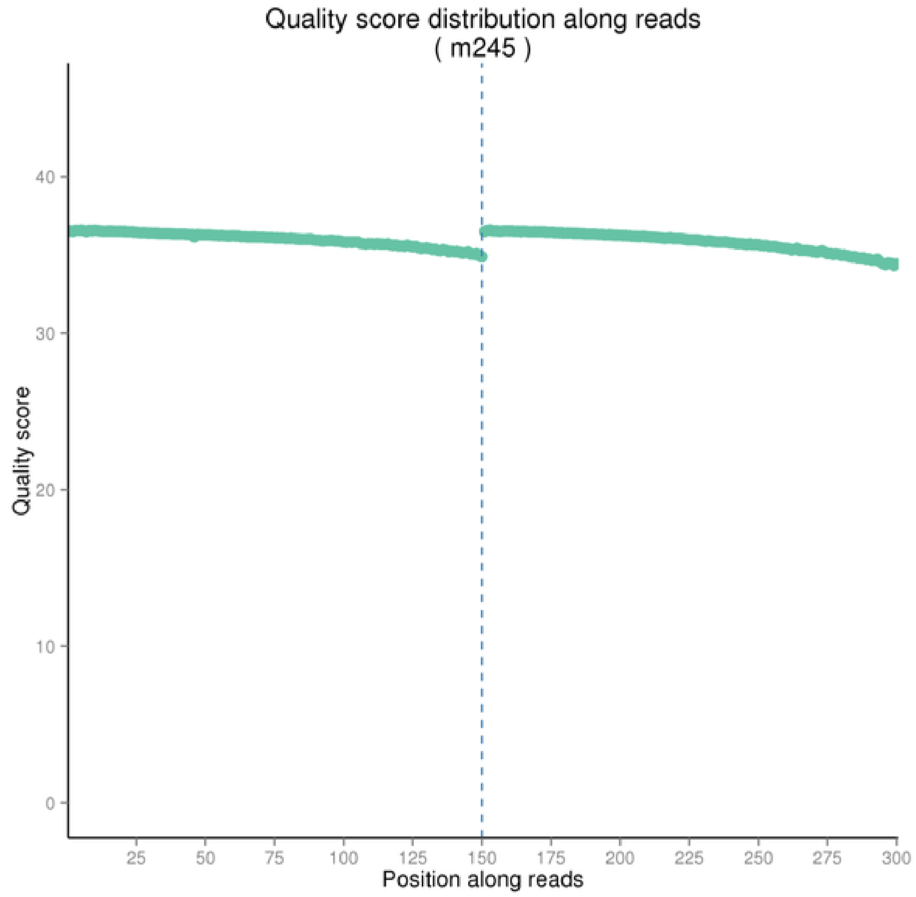

Figure S15. Quality score distribution of Illumina RNA sequencing reads (m245-Foot RNA).

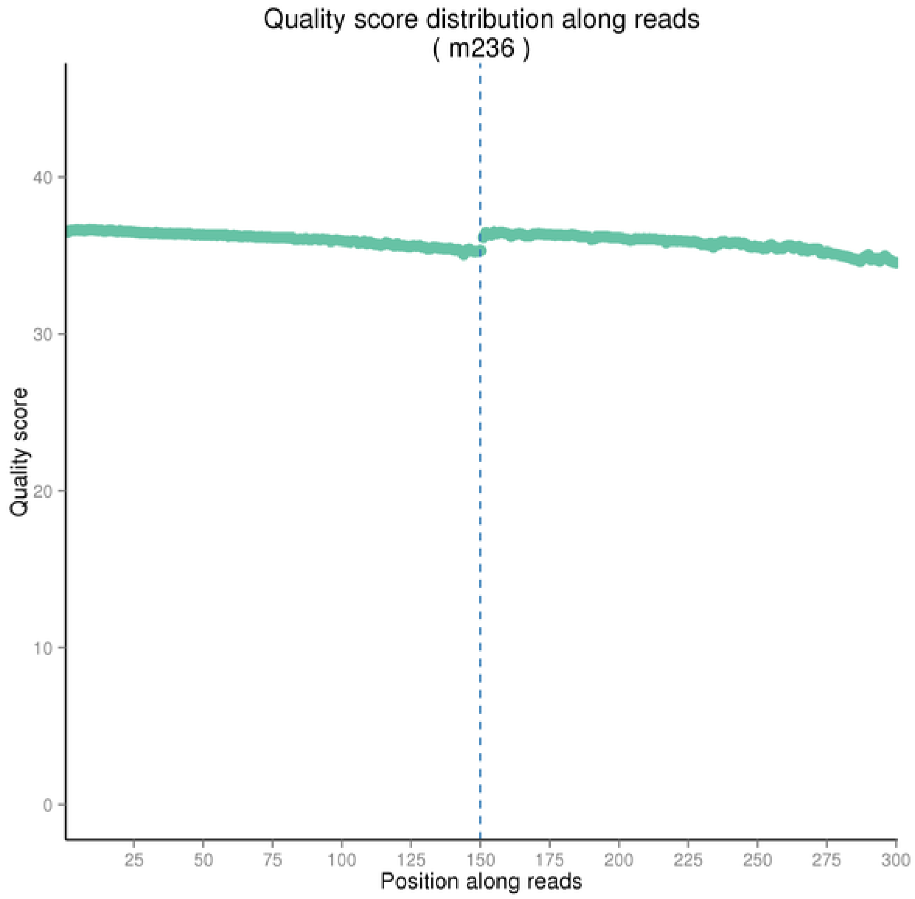

Figure S16. Quality score distribution of Illumina RNA sequencing reads (m236 Mantle RNA).

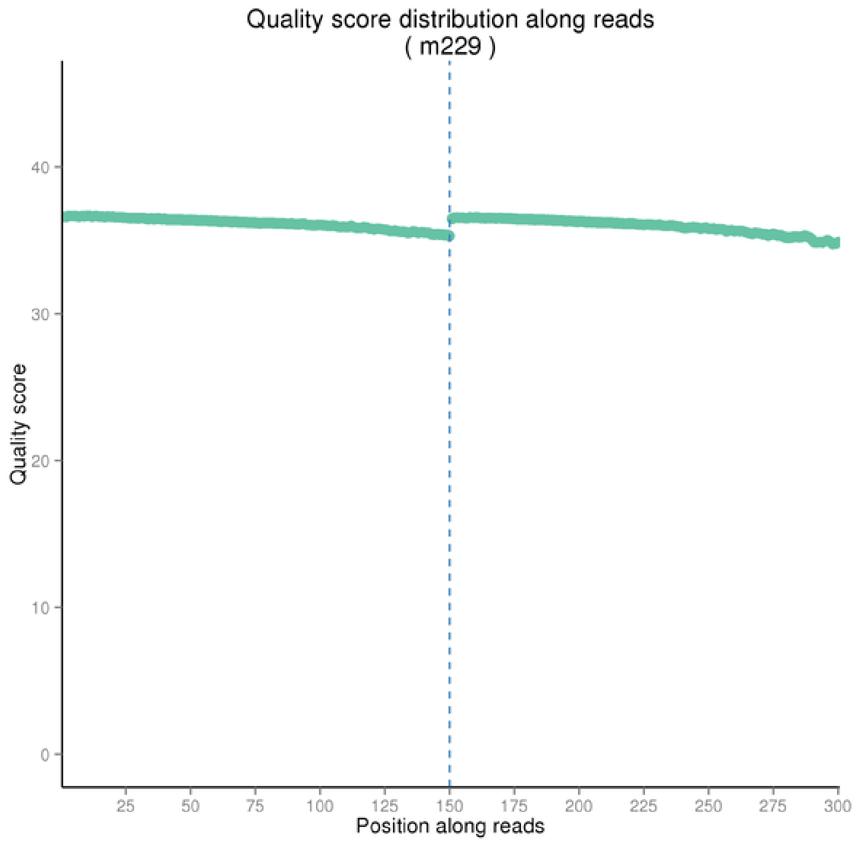

Figure S17. Quality score distribution of Illumina RNA sequencing reads (m229 Digestive Gland RNA).

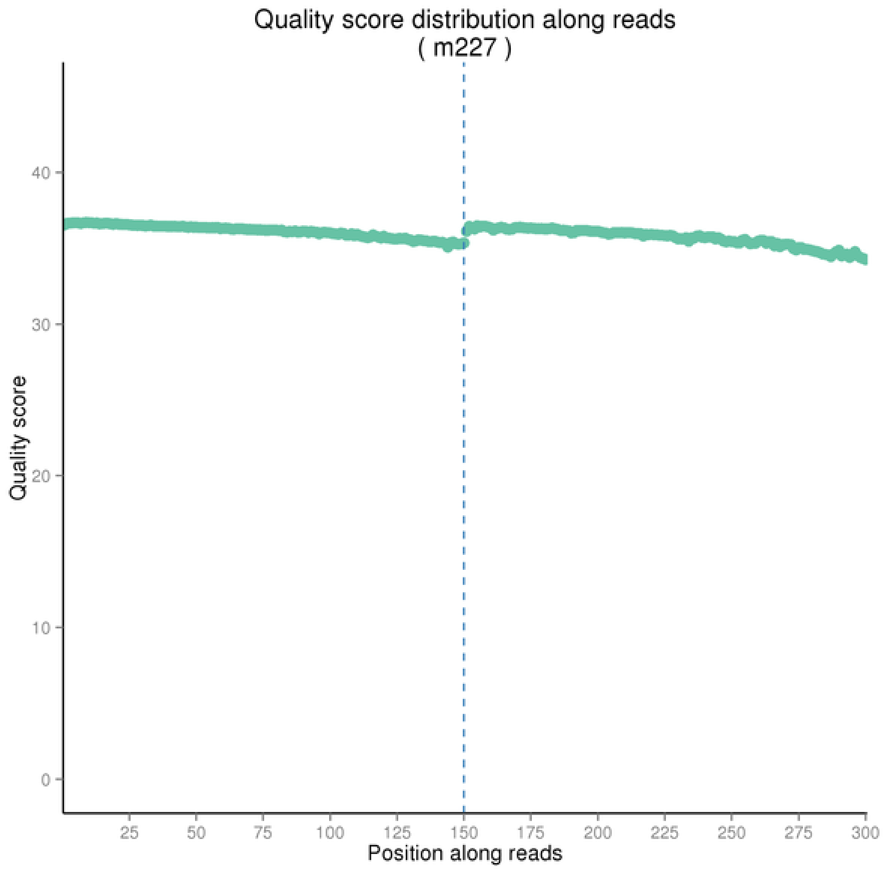

Figure S18. Quality score distribution of Illumina RNA sequencing reads (m227 Gill RNA).

Method S3.3. Distribution of Illumina RNA sequencing error rate

The error rate is shown in Figures S19 (m245-Foot RNA), S20 (m236 Mantle RNA), S21 (m229 Digestive Gland RNA), and S22 (m227 Gill RNA), where the base position along the reads is on the horizontal axis and the single base error rate is on the vertical axis.

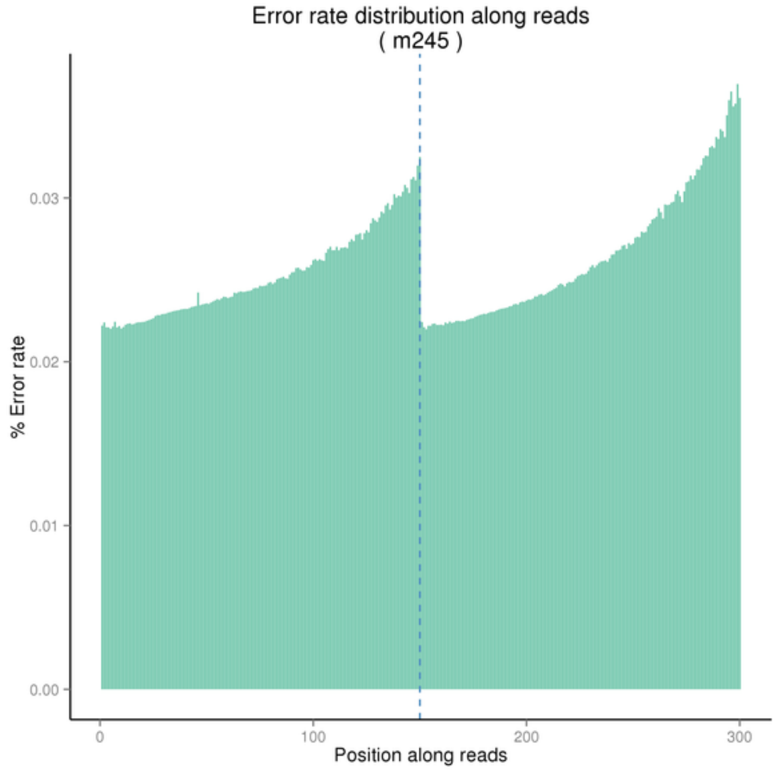

Figure S19. Illumina RNA sequencing error rate by read base position (m245-Foot RNA).

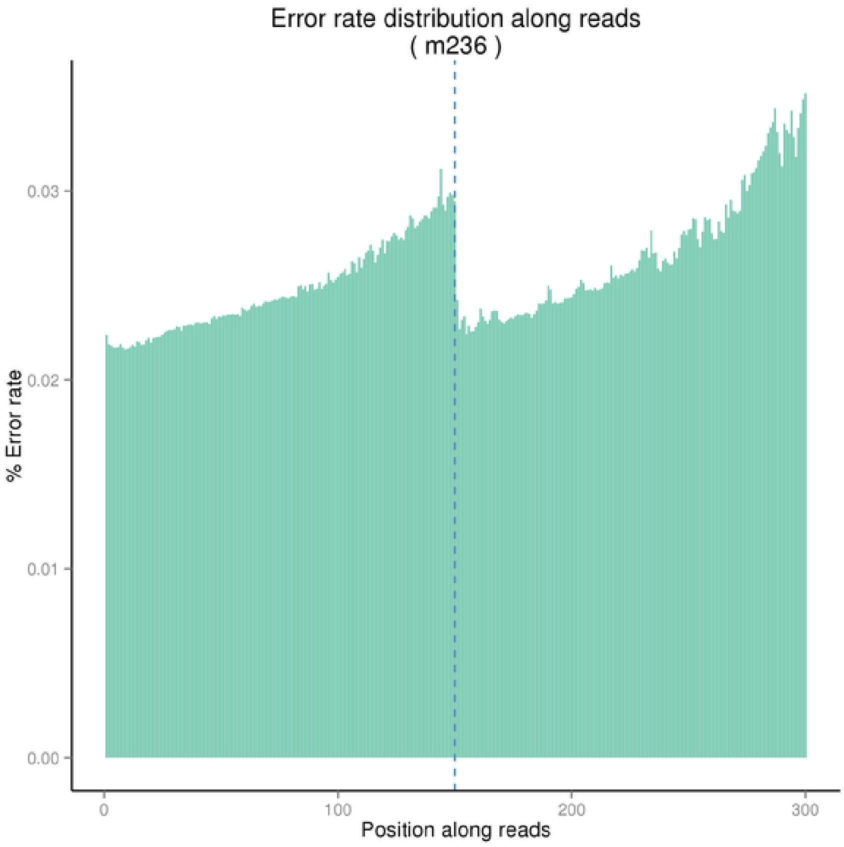

Figure S20. Illumina RNA sequencing error rate by read base position (m236 Mantle RNA).

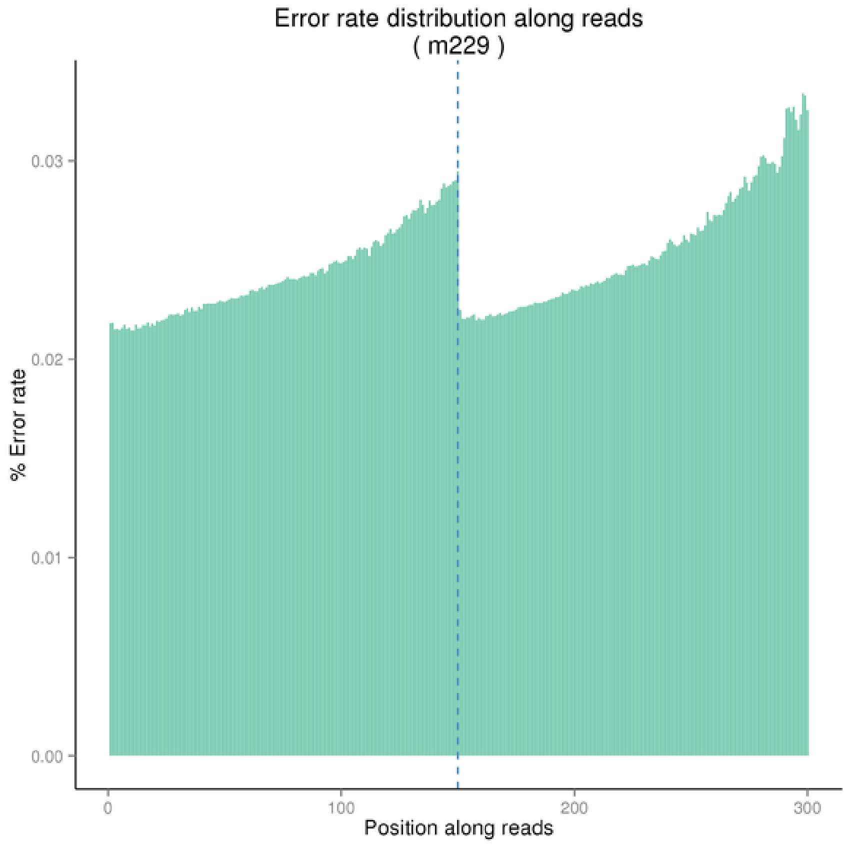

Figure S21. Illumina RNA sequencing error rate by read base position (m229 Digestive Gland RNA).

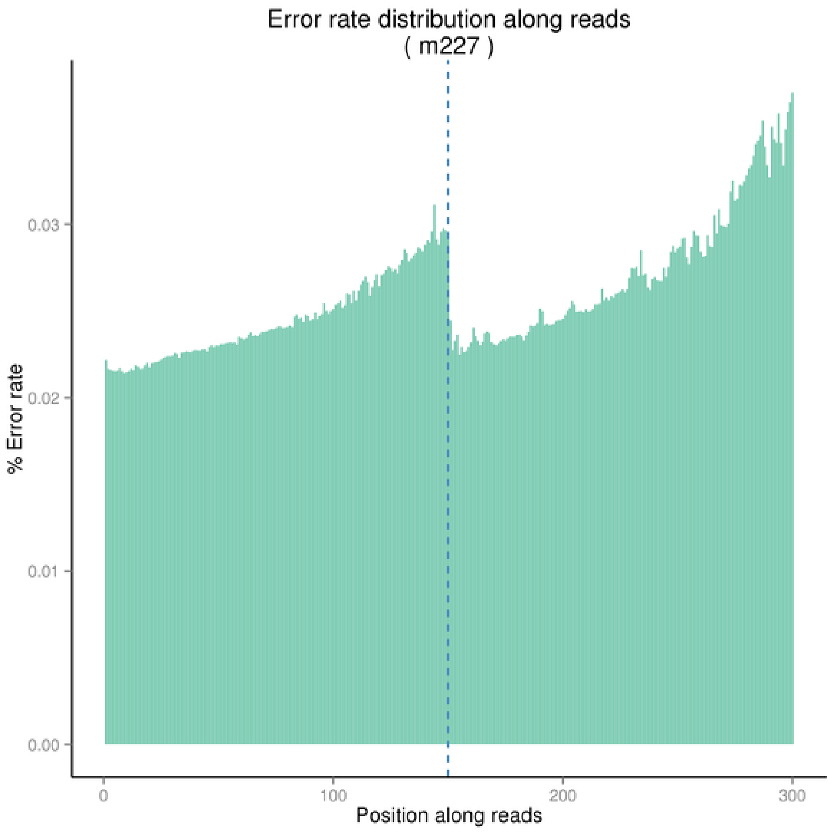

Figure S22. Illumina RNA sequencing error rate by read base position (m227 Gill RNA).

Method S3.4. Distribution of Illumina RNA sequencing A/T/G/C Bases

The distribution of GC content is shown in Figures S23 (m245-Foot RNA), S24 (m236 Mantle RNA), S25 (m229 Digestive Gland RNA) and S26 (m227 Gill RNA) where the base position along reads is on the horizontal axis and the percentage of each base is on the vertical axis.

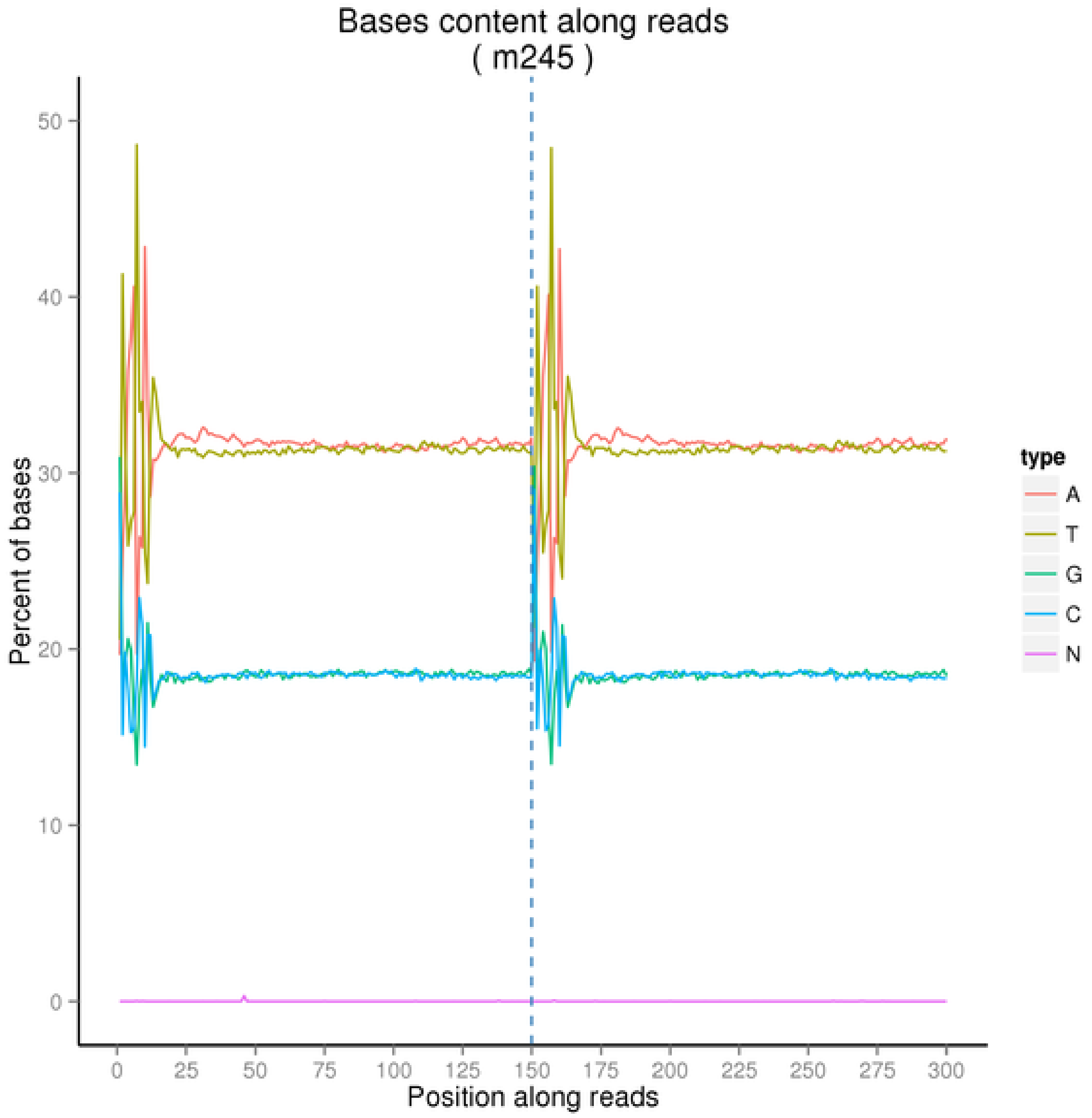

Figure S23. Illumina RNA sequencing A/T/G/C distribution by read base position (m245-Foot RNA).

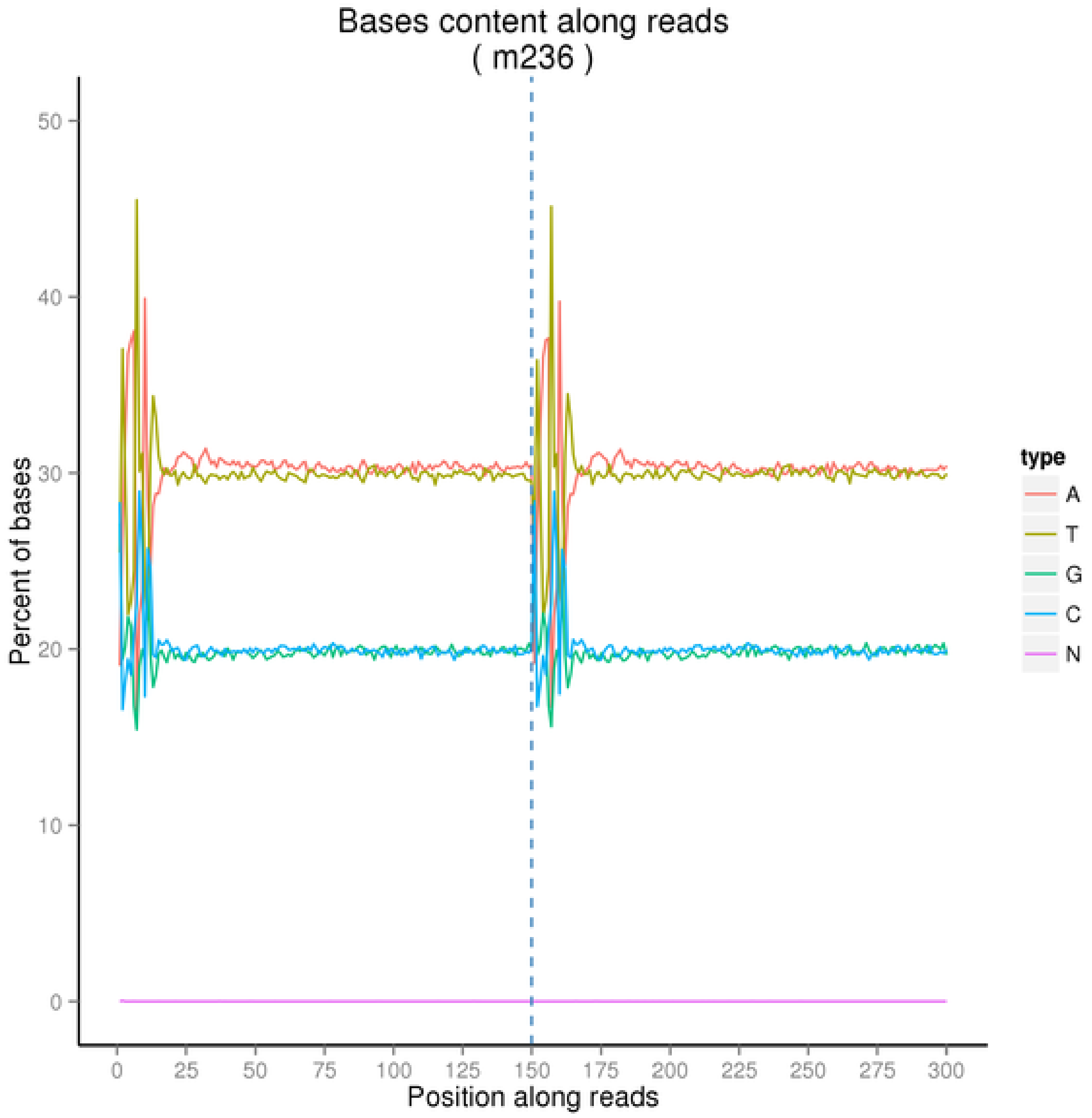

Figure S24. Illumina RNA sequencing A/T/G/C distribution by read base position (m236 Mantle RNA).

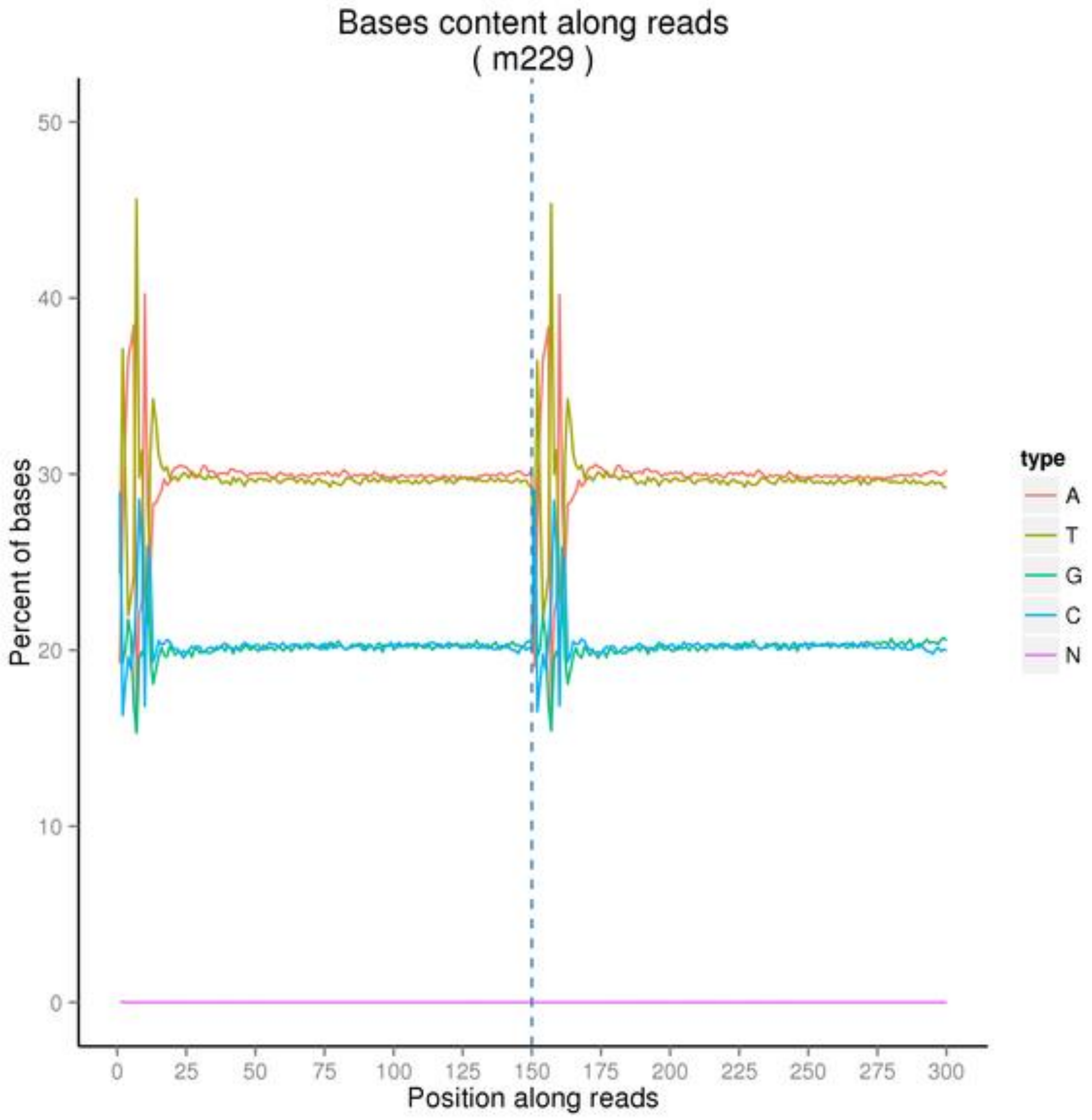

Figure S25. Illumina RNA sequencing A/T/G/C distribution by read base position (m229 Digestive Gland RNA).

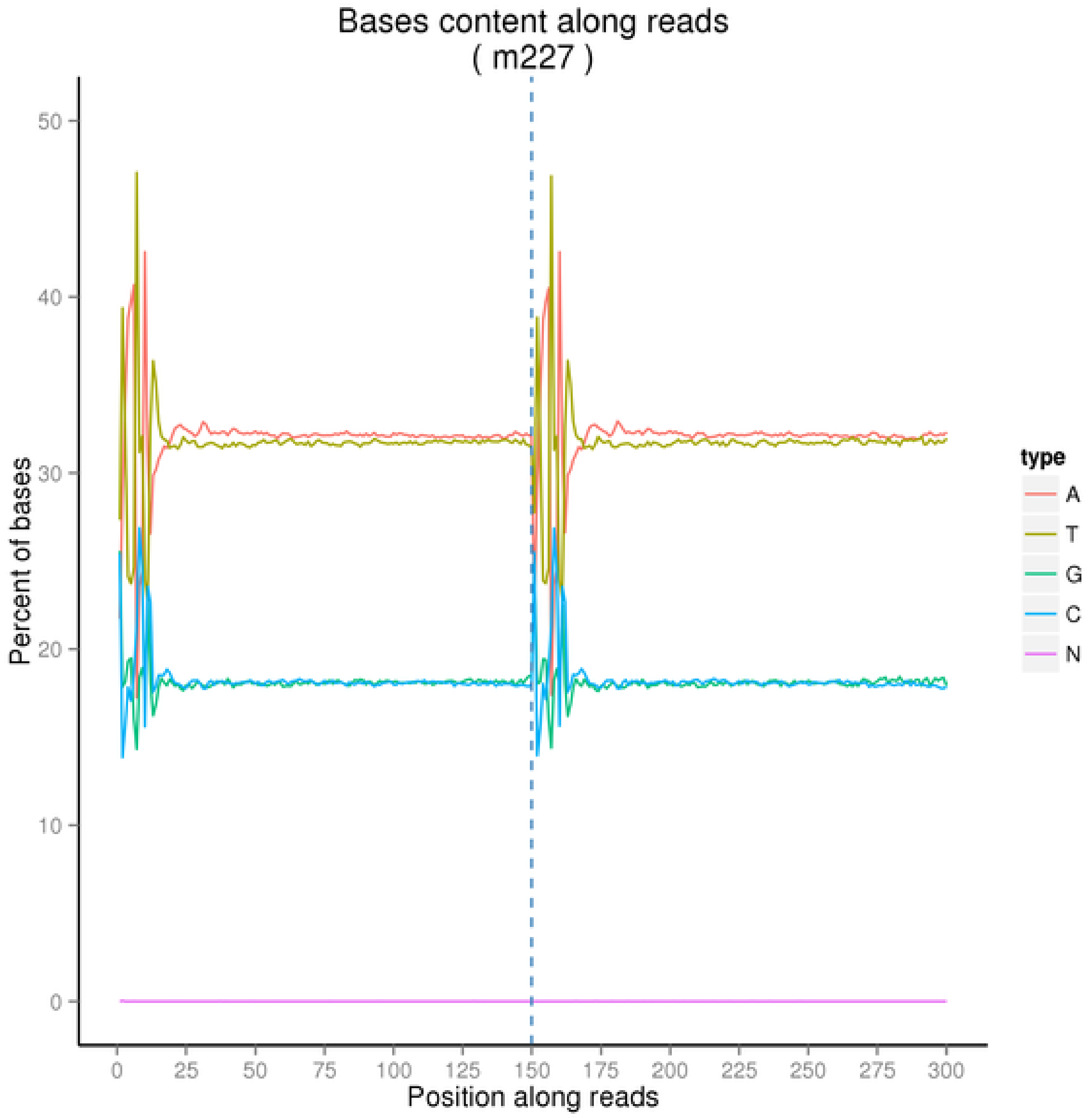

Figure S26. Illumina RNA sequencing A/T/G/C distribution by read base position (m227 Gill RNA).

Method S3.5. Illumina RNA Raw Data Filtering

The sequenced reads (raw reads) were filtered to exclude low quality reads and adapters as follows:

(1) Remove reads containing adapters.

(2) Remove reads containing N > 10% (N represents a base that cannot be determined).

(3) Remove reads containing low quality (Qscore<= 5) bases.

Sequences of adapters

Adapter:

5'-AGATCGGAAGAGCGTCGTGTAGGGAAAGAGTGTAGATCTCGGTGGTCGCCGTATCATT-3'

3'

Adapter:

5'-GATCGGAAGAGCACACGTCTGAACTCCAGTCACGGATGACTATCTCGTATGCCGTCTTCTGCTTG-3'

Raw read filtering results are shown in shown in Figures S27 (m245-Foot RNA), S28 (m236 Mantle RNA), S29 (m229 Digestive Gland RNA) and S30 (m227 Gill RNA) based on:

(1) Adapter related: (reads containing adapter) / (total raw reads)

(2) Containing N: (reads with more than 10% N) / (total raw reads)

(3) Low quality: (reads of low quality) / (total raw reads)

(4) Clean reads: (clean reads) / (total raw reads)

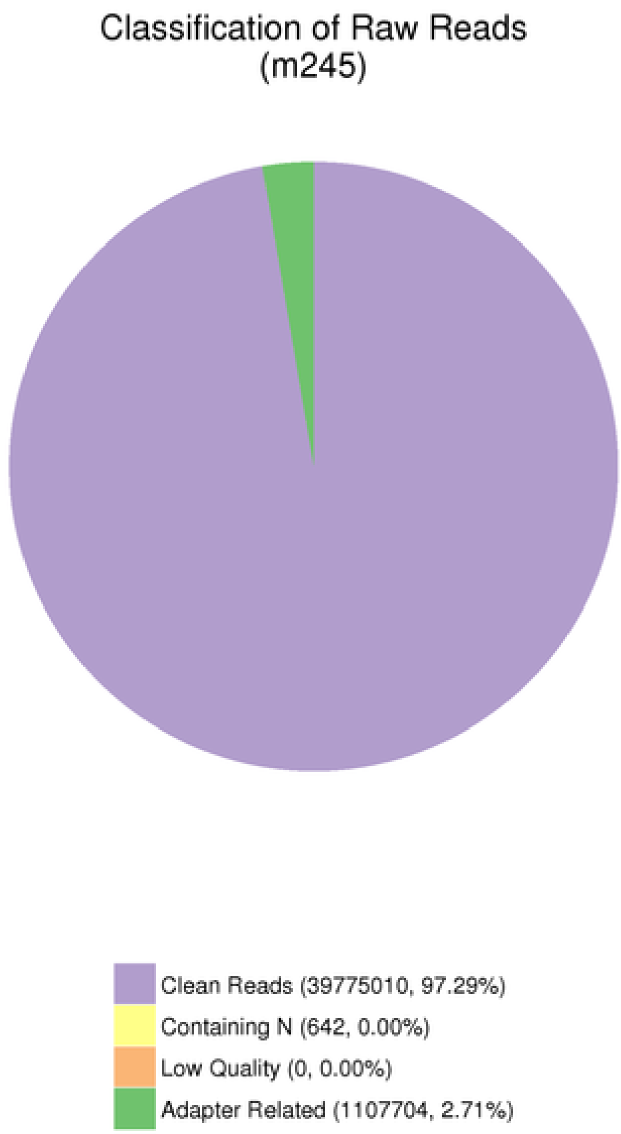

Figure S27. Illumina RNA raw read filtering results (m245-Foot RNA).

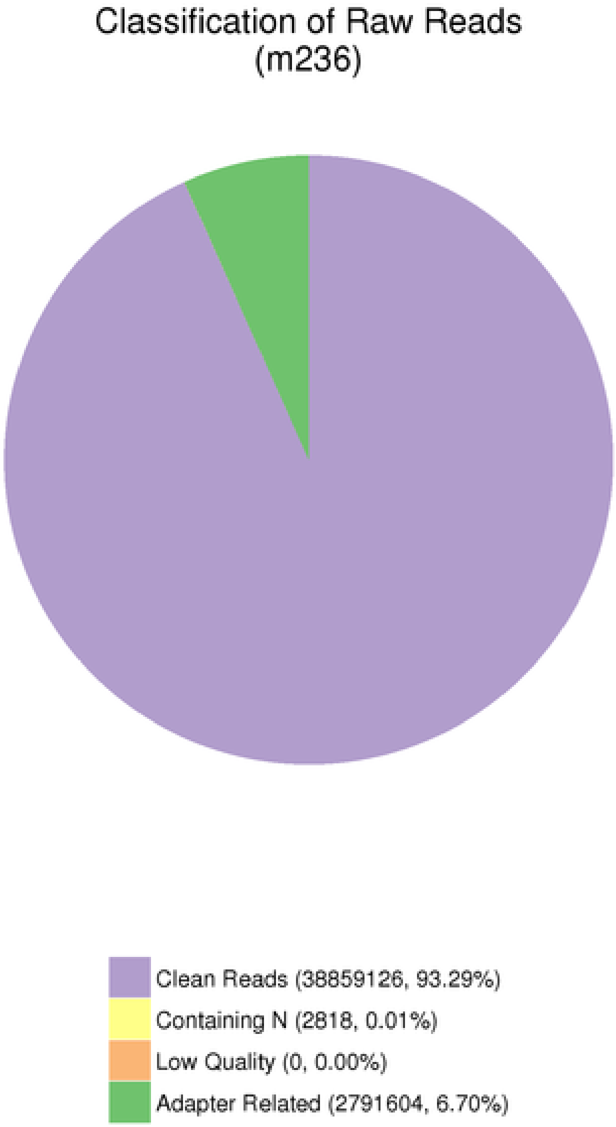

Figure S28. Illumina RNA raw read filtering results (m236 Mantle RNA).

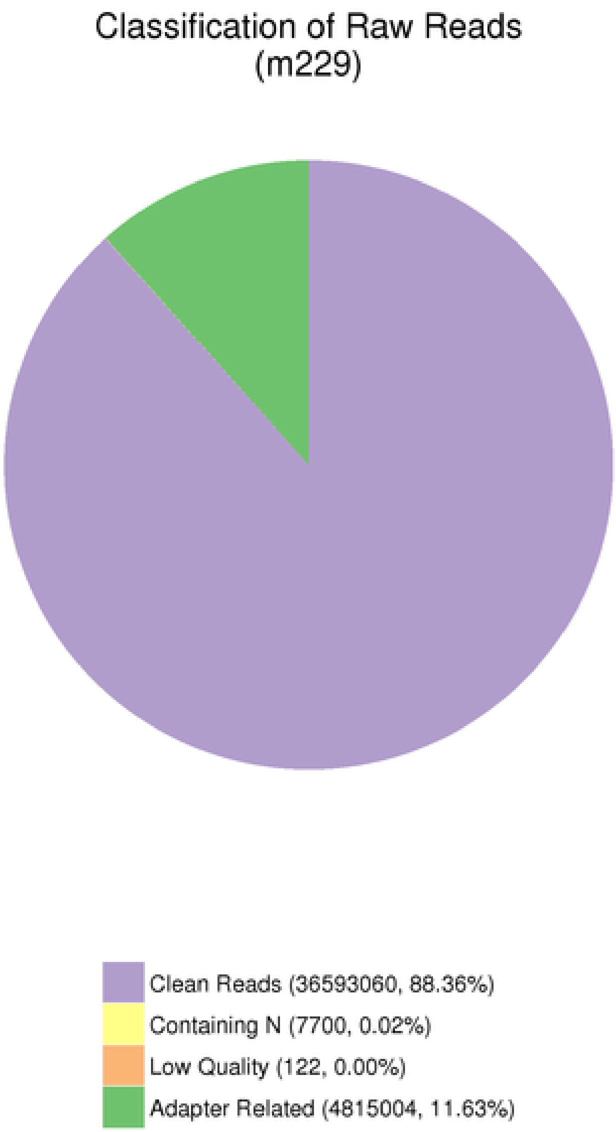

Figure S29. Illumina RNA raw read filtering results (m229 Digestive Gland RNA).

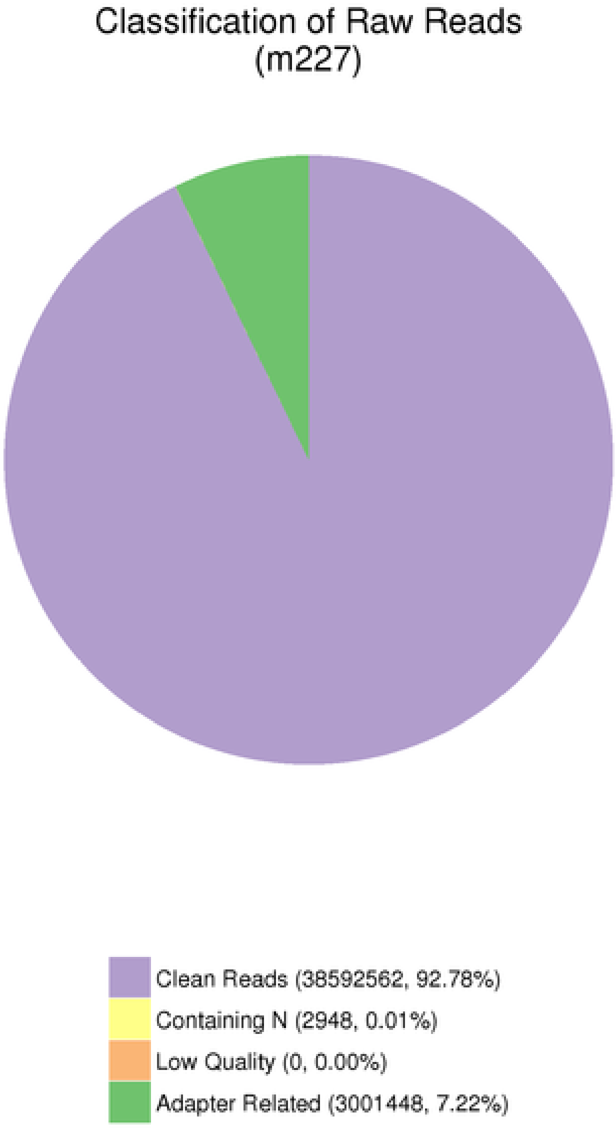

Figure S30. Illumina RNA raw read filtering results (m227 Gill RNA).

Method S3.6. Summary of Illumina RNA sequencing data

The total output of raw data from the sequencer was 24.7 Gb. The detail statistics for the quality of sequencing data are shown in Table S4**.**

.

Table S4. RNA Sequencing Data Quality Summary.

Sample	Raw reads(#)	Raw data	Effective(%)	Error(%)	Q20(%)	Q30(%)	GC(%)
m245-Foot	40883356	6.1	97.29	0.03	97.79	93.39	37.10
m236-Mantle	41653548	6.2	93.29	0.03	97.73	93.53	39.93
m227-Dig.Gl.	41596958	6.2	92.78	0.03	97.70	93.40	36.35
m229- Gill	41415886	6.2	88.36	0.03	97.85	94.15	40.53

Raw data: (Raw reads) * (sequence length), calculated in Gigabases.

Effective: (Clean reads/Raw reads)*100%

Error: base error rate

Q20, Q30: (Base count of Phred value > 20 or 30) / (Total base count)

GC: (G & C base count) / (Total base count)

Method S4. Genome Survey

Method S4.1 K-mer Analysis

Before assembly, genome size was estimated by kmer analysis using Kmer=17. The estimated genome size before and after revision, heterozygous rate, and repeat rate are shown in Table S5. K-mer distribution diagram is shown in Figure S31. The peak of the k-mer depth distribution is depth 38. The genome size estimated by the formula: Genome Size=K-mer_num/Peak_depth) is 1,763.52 Mbp, the revised genome size is 1,751.71 Mbp. The heterozygous rate is 1.15%, and the repeat content is 67.66%.

Table S5. Kmer analysis summary.

Kmer	Depth	n_kmer	Genome size (Mb)	Revised Genome size (Mb)	Heterozygous rate (%)	Repeat rate (%)
17	38	67,013,742,156	1,763.52	1,751.71	1.15	67.66

(1) K-mer: Selected K-mer length.

(2) Depth: The expected value of K-mer depth.

(3) n_K-mer: The total number of K-mer from SOAPdenovo.

(4) Genome size (M): The genome size in Mb estimated by formula: Genome Size=K-mer_num/Peak_depth.

(5) Revised Genome size (M): Revised genome size after error correction from wrong K-mer.

(6)Heterozygous ratio: The percent of heterozygous positions.

(7) Repeat: Calculated by the percentage of K-mer numbers after 1.8-fold of the main peak of total K-mer numbers.

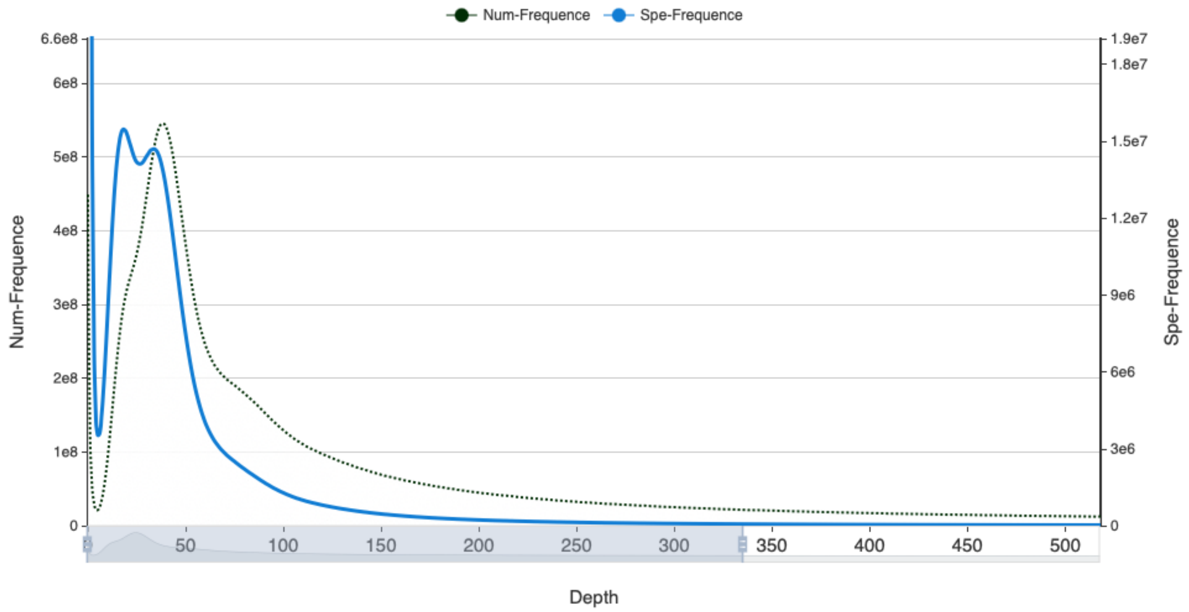

Figure S31. K-mer distribution. X-coordinate is K-mer depth. Y-coordinate is the frequency of each K-mer depth.

Method S5. Primary Genome Assembly

Method S5.1. Overall Strategy

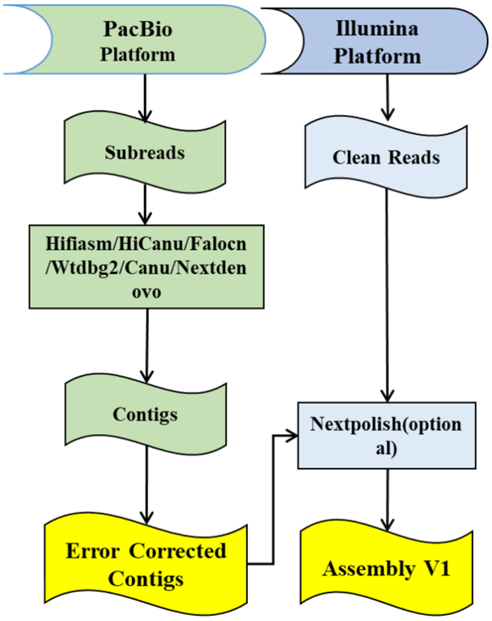

Figure S32. Genome assembly strategy.

Method S5.2. Data correction

Under high sequencing depth, k-mers of low frequency were regarded as results of sequencing error. In data correction, the correction includes establishing a k-mer frequency table, setting high frequency and low frequency thresholds, and conversion of low frequency k-mers to high frequency k-mers by changing specific bases in reads.

Method S5.3. Scaffolding and contig assembly

All reads were mapped to primary contigs, and contigs were further assembled to scaffolds on the basis of read length and insert size information. The distributions of contig

length and number with coverage depth are shown in Figures S33 and S34. The correlation of GC content (35.87%) and sequencing depth of contigs are in Figure S35.

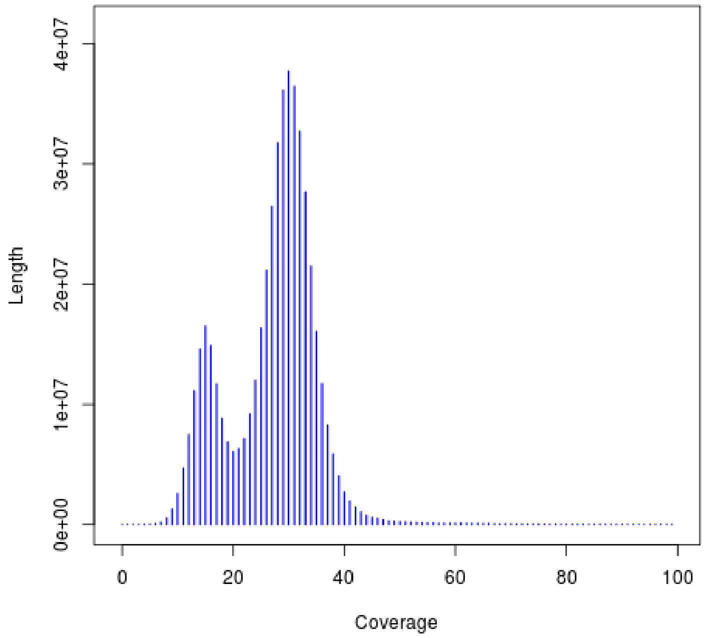

Figure S33. Distribution of the contig length and coverage depth.

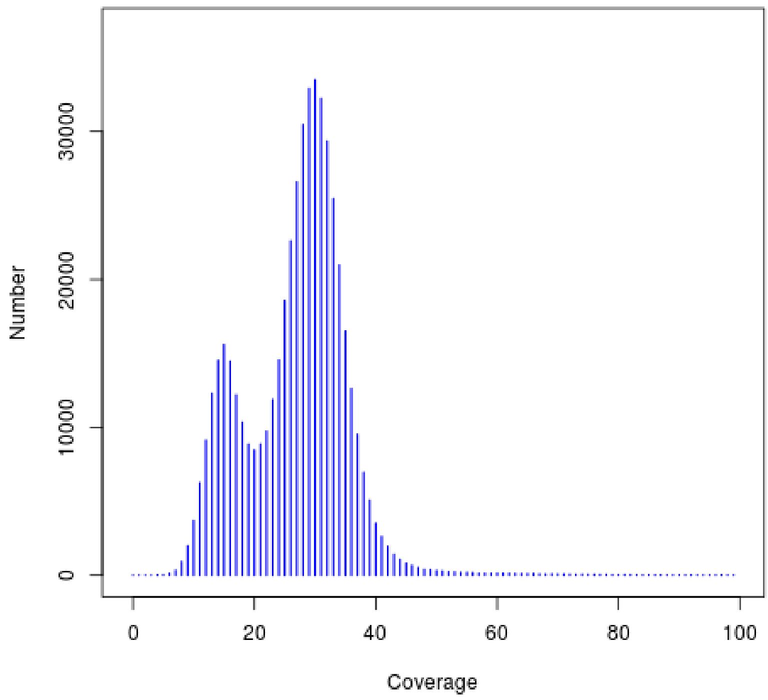

Figure S34. Distribution of the contig numbers and coverage depth.

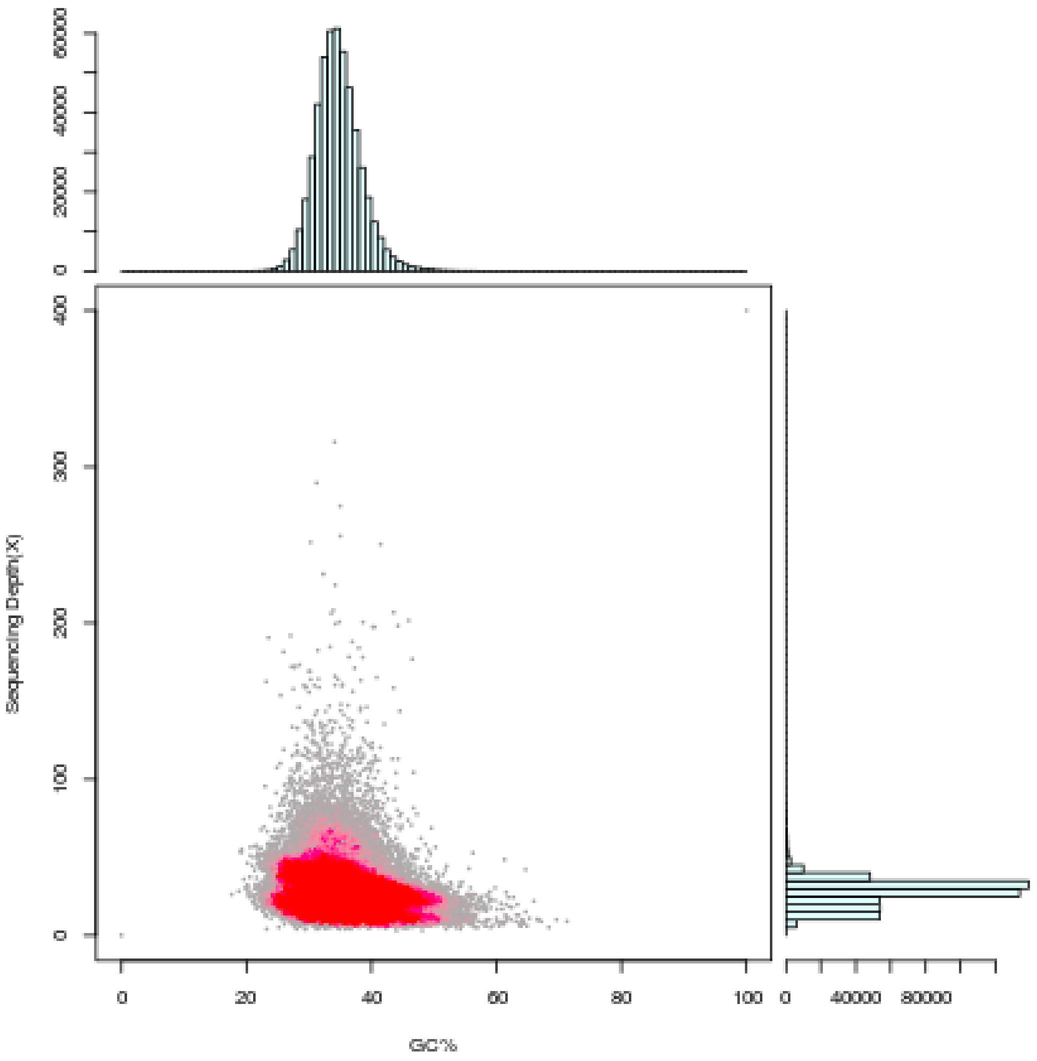

Figure S35. Correlation of GC content and sequencing depth of contigs. X-coordinate is GC content, and Y-coordinate is sequencing depth. Bars in the right side show the distribution of contig depth. Bars on top show the distribution of GC contents. Red color indicates higher density in this scatter plot.

Method S5.5. Genome Assembly Assessment

Sequence coverage statistics are shown in Table S6 and Figure S36. Total sequencing data volume was 437 Gb, and the coverage was 247.80X (calculated by estimated genome size 1763.52M in genome survey). GC content is shown in Table S7.

Table S6. Sequence coverage.

Library	Total data (G)	Sequence coverage (X)
Illumina reads	75	42.53
PacBio reads	437	247.80
Total	512.00	290.33

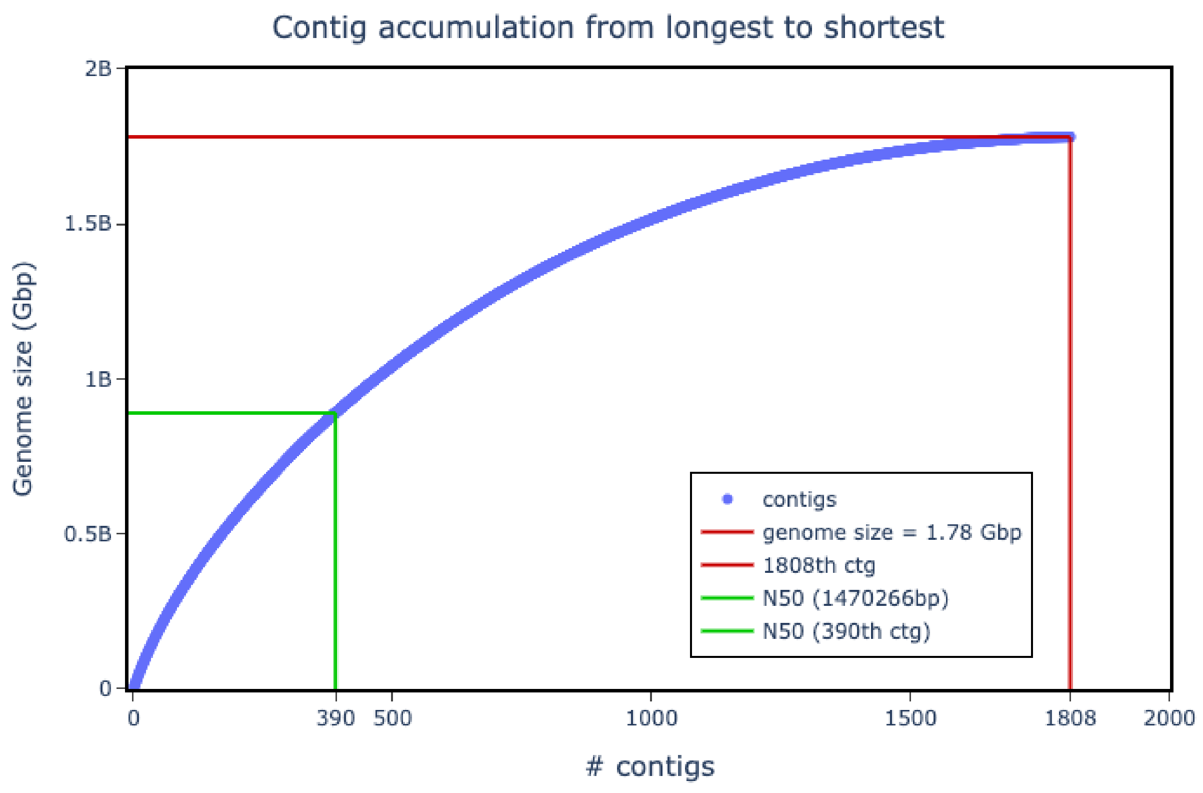

Figure S36. Cumulative contig summary (shortest to longest).

Table S7. Genome A/T/G/C contents.

	Number (bp)	% of genome
A	574,265,920	32.24
T	574,210,294	32.24
C	316,441,011	17.77
G	316,235,559	17.75
N	0	0.00
total	1,781,152,784	--
GC	632,676,570	35.52

Method S5.6. BUSCO assessment

Genome completeness was evaluated by combining BUSCO (Benchmarking Universal Single-Copy Orthologs：http://busco.ezlab.org/) with tools including tblastn, augustus, and hmmer. *Arctica islandica* genome BUSCO assessment statistics for 978 total BUSCO groups searched are listed in Table S8 and represented in Figure S37.

Table S8. BUSCO assessment statistics.

**BUSCO Group**	**Percentage of 278**
Complete BUSCOs	92.7%
Complete and single-copy BUSCOs	84.2%
Complete Duplicated BUSCOs	8.5%
Fragmented BUSCOs	1.6%
Missing BUSCOs	5.7%

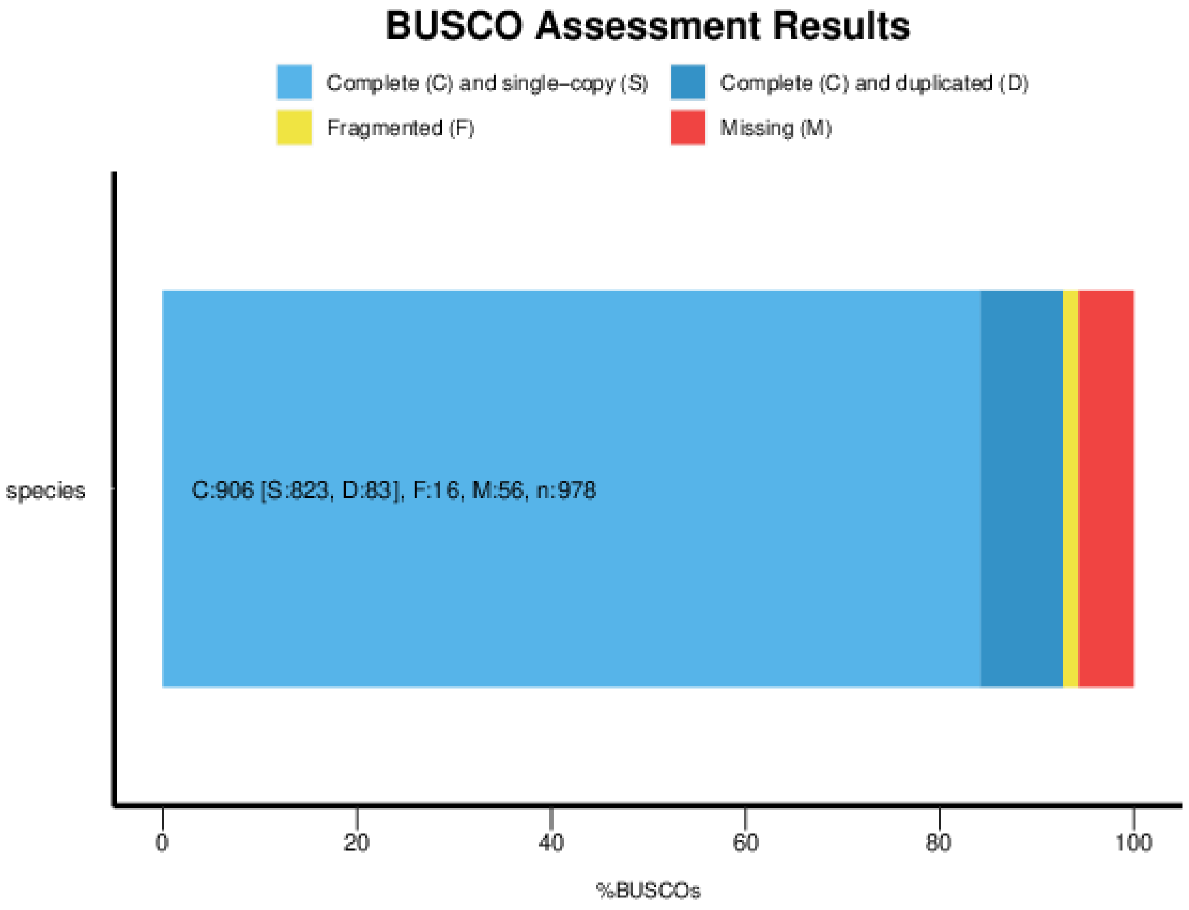

Figure S37. BUSCO assessment statistics.

Method S5.7. CEGMA assessment

Genome completeness was also evaluated by combining CEGMA (Core Eukaryotic Genes Mapping Approach：http://korflab.ucdavis.edu/datasets/cegma/), which selects 248 conserved genes in 6 eukaryotes as core genes, with tools including tblastn, genewise and geneid. *Arctica islandica* genome CEGMA assessment statistics are listed in Table S9.

Table S9. CEGMA assessment statistics.

Complete		Complete + Partial	
# Proteins	% Completeness	# Proteins	% Completeness
209	84.27	228	91.94

Complete： core gene >70% core gene sequences are assembled.

Complete + partial: core gene sequences are either completely or partially assembled.

#Proteins： number of assembled core genes.

% completeness： percentage of assembled core genes in 248 core genes.

.

Method S5.8. Sequence consistency assessment

Illumina short reads were mapped to the assembled genome by BWA (http://bio-bwa.sourceforge.net/) and the mapping rate/coverage/depth are listed in Table S10. The read mapping rate was 97.35%, and coverage is 94.45%, indicating high consistency between the assembly and the reads. The sequencing depth distribution is shown in Figure S38 where the sequencing depth is on the X axis and the proportion of bases in the genome on the Y-axis.

Table S10. Arctica genome read mapping rate/coverage/depth statistics.

Mapping rate (%)	97.35
Average sequencing depth	39.94
Coverage (%)	94.45
Coverage at least 4X (%)	92.56
Coverage at least 10X (%)	89.81
Coverage at least 20X (%)	80.66

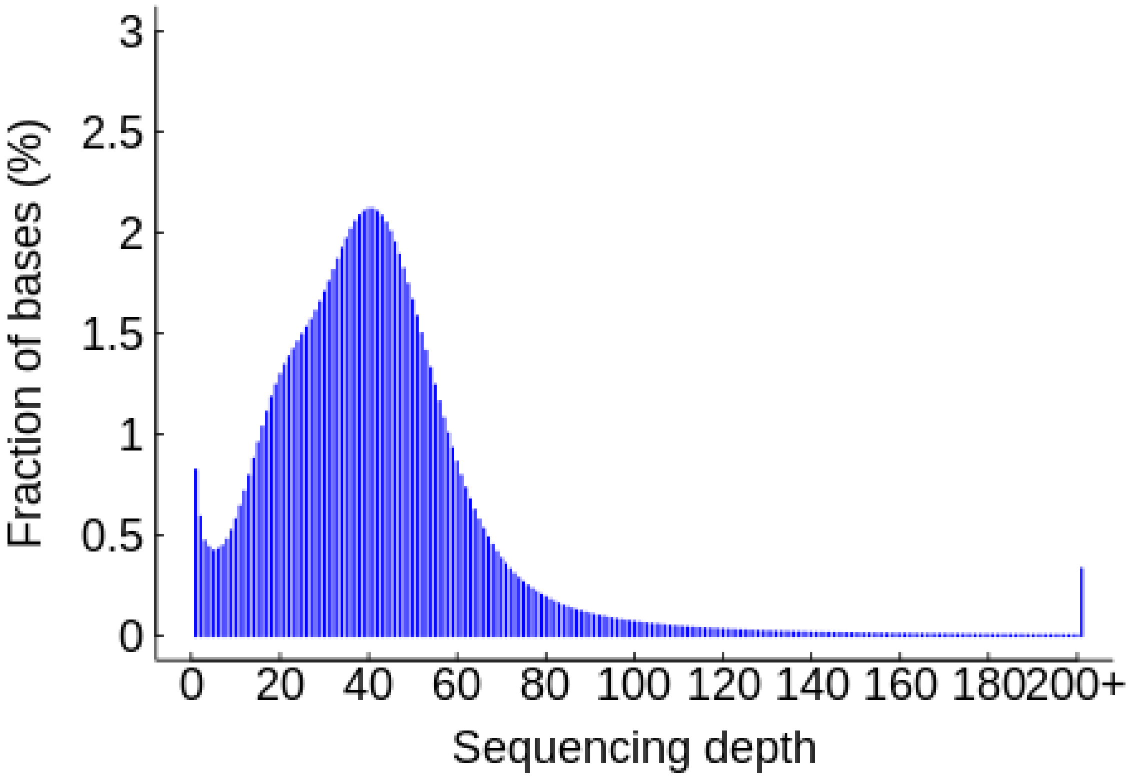

Figure S38. Sequencing depth distribution.

For the assembled genome, 10k sliding windows were used to calculate GC contents and average sequencing depth to check for possible GC bias contamination during sequencing using Blobtools (v.1.1.1). Unsegregated GC distribution indicates clean sequencing process. In brief, GC contents are mainly around 35.52, and the scatter plot (Figure S39) shows no significant segregation, indicating no contamination of the genome.

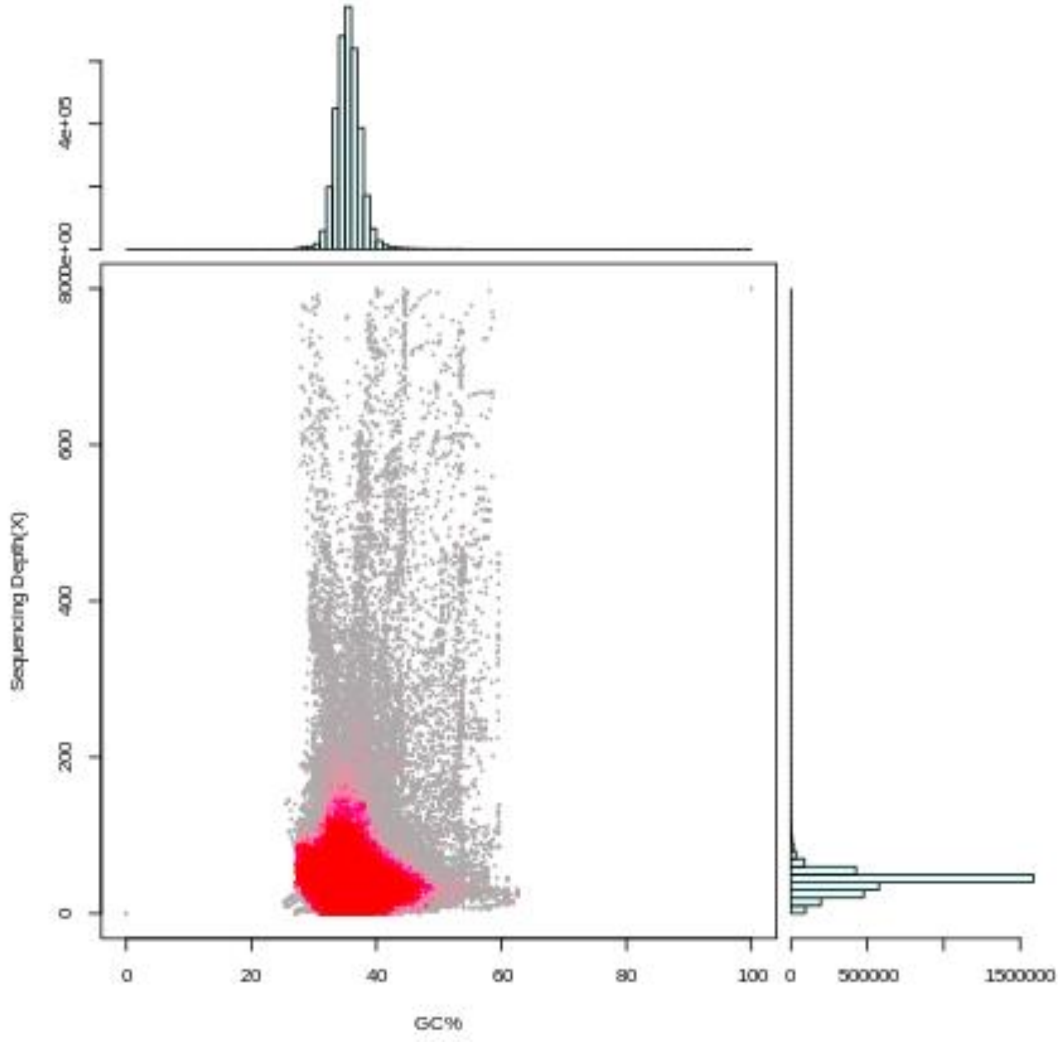

Figure S39. Sequencing depth distribution versus GC content. Main: X-axis: GC contents; y-axis: sequencing depth. Upper: GC content distribution. Lower right: sequencing depth distribution.

Single Nucleotide Polymorphisms (SNP), i.e., single nucleotide variation, were used along with including SAMTools(http://samtools.sourceforge.net/) to order and deduplicate BWA mapping results, call SNPs, and filter original results. Final SNP statistics are shown in

table S14. The heterozygosity SNP ratio was 0.571779%, and homozygous SNP is 0.048273%. Lower homozygous SNP ratios indicate higher assembly accuracy.

Table S11. SNP statistics.

	Number	Percentage
All SNP	9,923,749	0.620053%
Heterozygous SNP	9,151,147	0.571779%
Homozygous SNP	772,602	0.048273%

Method S5.9. Hi-C Library Construction

High-throughput chromosome conformation capture or Hi-C was attempted to improve assembly accuracy. In this technique, 50 mg - 200 mg of tissue that had been snap frozen in dry ice and stored at -80°C was ground to a fine powder in liquid nitrogen using a mortar and pestle. The powdered tissue was then incubated with cross-linking solution for 15 minutes at RT. After quenching, the cells were pelleted by centrifugation, then lysed to release chromatin. Following restriction digestion and end repair with biotin incorporation, proximal ends were ligated with T4 DNA ligase. The cross-links were then reversed and the DNA purified for library preparation for Illumina sequencing. The Proximo Hi-C (Animal) kit was used on gill, adductor muscle, mantle tissue, and foot muscle. The quality of the libraries isolated from these tissues is shown in Figure S40. None of them were considered to pass QC due to wide fragment size distribution. All four libraries were just above the minimum concentrations required for sequencing. None of the sequencing was considered adequate due to hicup evaluation failing to meet the effective criterion of greater than 20% and the ratio of valid pairs greater than 75%.

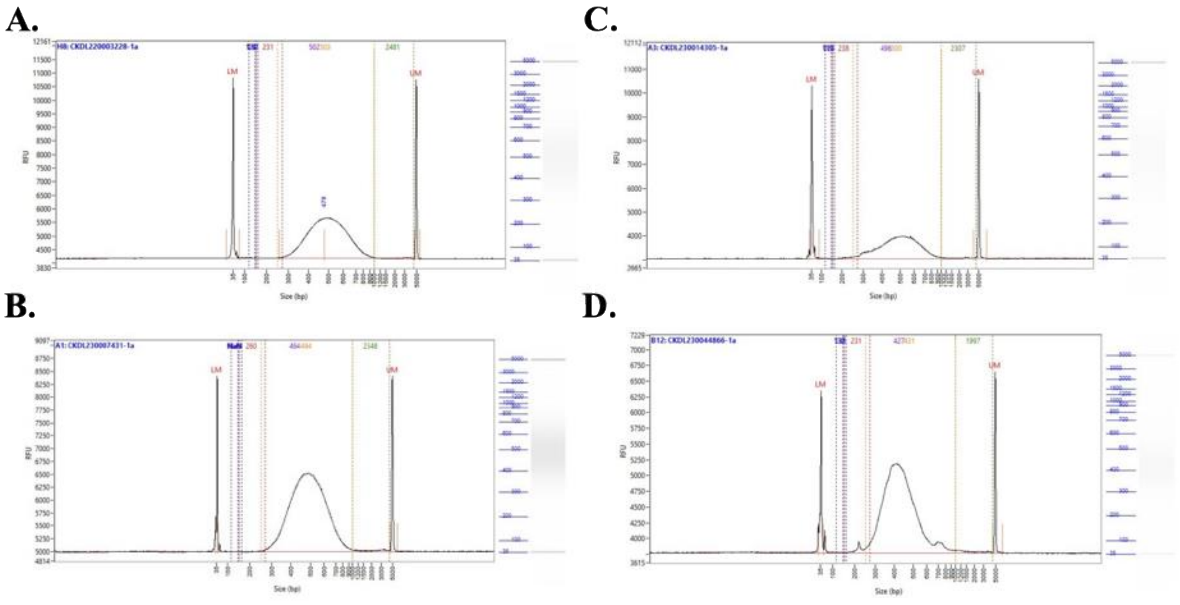

Figure S40. Bioanalyzer results for Hi-C libraries prepared from 4 tissues. A. Gill. B. Adductor muscle. C. Mantle. D. Foot muscle.

Method S6. Genome Annotation

Method S6.1. Annotation Strategy

Genome annotation includes repeat sequence annotation, gene annotation (including gene structure annotation and gene function annotation) and non-coding RNA (ncRNA) annotation, as shown in the main text Figure 2.

Method S6.2. Gene function annotation

Gene structure annotations were aligned with known protein databases including SwissProt (http://www.uniprot.org/), Nr (http://www.ncbi.nlm.nih.gov/protein), Pfam (http://pfam.xfam.org/), KEGG (http://www.genome.jp/kegg/) and InterPro (https://www.ebi.ac.uk/interpro/) to generate gene function information.

Non-coding RNAs including tRNA, rRNA, miRNA and snRNA were also annotated. tRNAs were found by tRNAscan-SE (http://lowelab.ucsc.edu/tRNAscan-SE/) based on structural features. rRNAs were found by blast . miRNAs and snRNAs were predicted by INFERNAL (http://infernal.janelia.org/, provided by Rfam) based on covariance models (CMs).

Method S6.3. Repeat annotation

Repeat sequences are mainly composed of tandem repeats and interspersed repeats. Tandem repeats include microsatellite repeats, minisatellite repeats, variable number tandem repeat (VNTR), etc. Interspersed repeats are also called transposable elements (TEs), and include DNA transposons and retrotransposons. Retrotransposons include LTR, LINE, SINE, etc. A combined strategy based on homology alignment and de novo search to identify the whole genome repeats were applied in our repeat annotation pipeline. Tandem repeats were extracted using TRF (http://tandem.bu.edu/trf/trf.html) by ab initio prediction. The Repbase (http://www.girinst.org/repbase) database employing RepeatMasker (http://www.repeatmasker.org/) software and its in-house scripts (RepeatProteinMask) with

default parameters was used to extract repeat regions. An ab initio prediction database was built for de novo repetitive elements by LTR_FINDER(http://tlife.fudan.edu.cn/ltr_finder/), RepeatScout (http://www.repeatmasker.org/), RepeatModeler (http://www.repeatmasker.org/RepeatModeler.html) with default parameters, then all repeat sequences with lengths >100bp and gap 'N' less than 5% constituted the raw transposable element(TE) library. A custom library (a combination of Repbase and our de novo TE library which was processed by uclust to yield a non-redundant library) was supplied to RepeatMasker for DNA-level repeat identification.

Repeats found in *de novo* prediction were integrated with homologous repeat sequences in Repbase, and annotated by RepeatMasker. Results show that [Arctica_Islandica] genome includes 61.59% repeat sequences, and annotation results are listed in Table S12.

Table S12. Repeat size and percentage of genome.

**Type**	**Repeat Size(bp)**	**% of genome**
Trf	204,792,110	11.50
Repeatmasker	1,029,426,374	57.80
Proteinmask	62,848,757	3.53
Total	1,097,030,666	61.59

Transposable element (TE) divergence annotated by RepeatMasker based on Repbase are shown in Figure 41. X-axis indicates divergence between TEs in *Arctica islandica* and corresponding sequences in Repbase. The Y-axis indicates the proportion of TE sequences in the genome under corresponding divergence. Colors indicate different TE types.

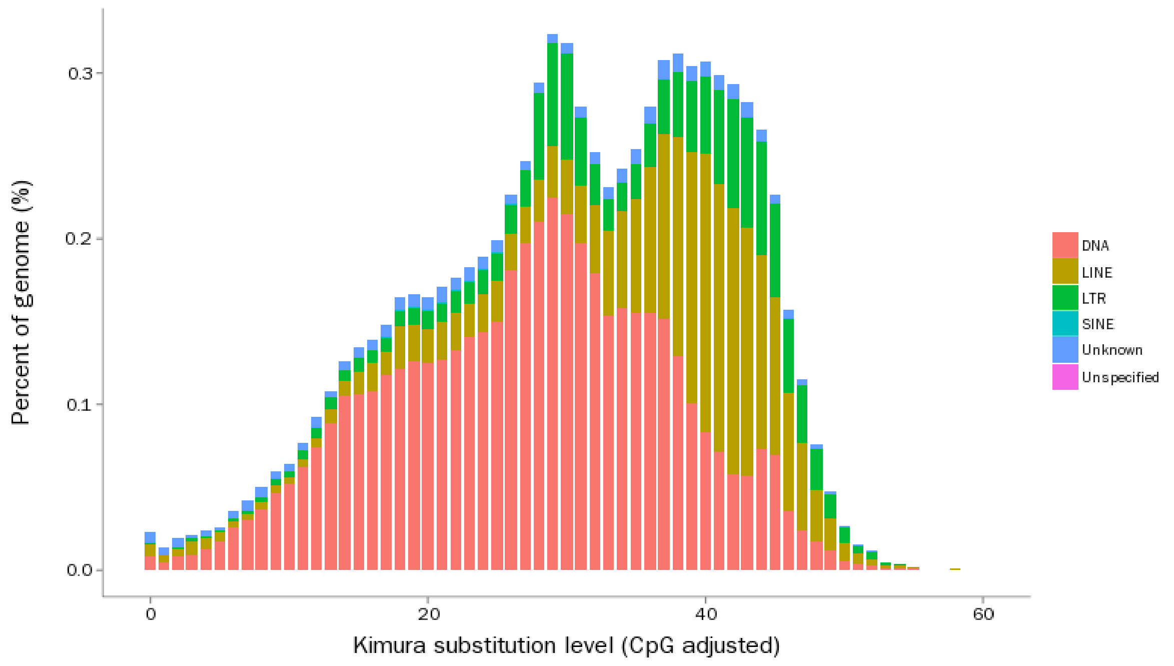

Figure S41. TE divergence annotated by RepeatMasker based on Repbase.

Method S6.4. Gene structure annotation

In *de novo* prediction, gene structure was predicted based on statistical features of genome sequence including codon frequency and exon-intron distribution as shown in Table S13.

In homologous prediction, species selected for homologous prediction included *Mercenaria mercenaria, Panopea generosa, Mactromeris polynyma, Tridacna gigas, Tridacna crocea, Serripes groenlandicus, Ruditapes philippinarum, Homo sapiens, and Margaritifera margaritifera.*

All predictions together with transcriptome alignment were integrated in EVidenceModeler (EVM, http://evidencemodeler.sourceforge.net/) to generate a non-redundant gene set, and annotation results are further corrected based on transcriptome data by PASA (http://pasa.sourceforge.net/) including addition of UTR and alternative splicing to

generate final results shown in Table S14. A Venn diagram of the evidence support for gene prediction is shown in Figure S43.

Table S13. De novo gene prediction based on 5 tools.

Gene set	Number	Average transcript length (bp)	Average CDS length (bp)	Average exons per gene	Average exon length (bp)	Average intron length (bp)
**Augustus**	50,427	12,168.09	1,175.29	5.00	234.91	2,746.00
**GlimmerHMM**	189,084	8,336.69	520.56	3.11	167.60	3,711.50
**SNAP**	118,657	4,668.13	615.78	3.19	193.12	1,851.58
**Geneid**	48,679	18,630.30	1,218.12	4.28	284.67	5,310.22
**Genscan**	48,507	22,367.19	1,346.33	5.46	246.38	4,708.41

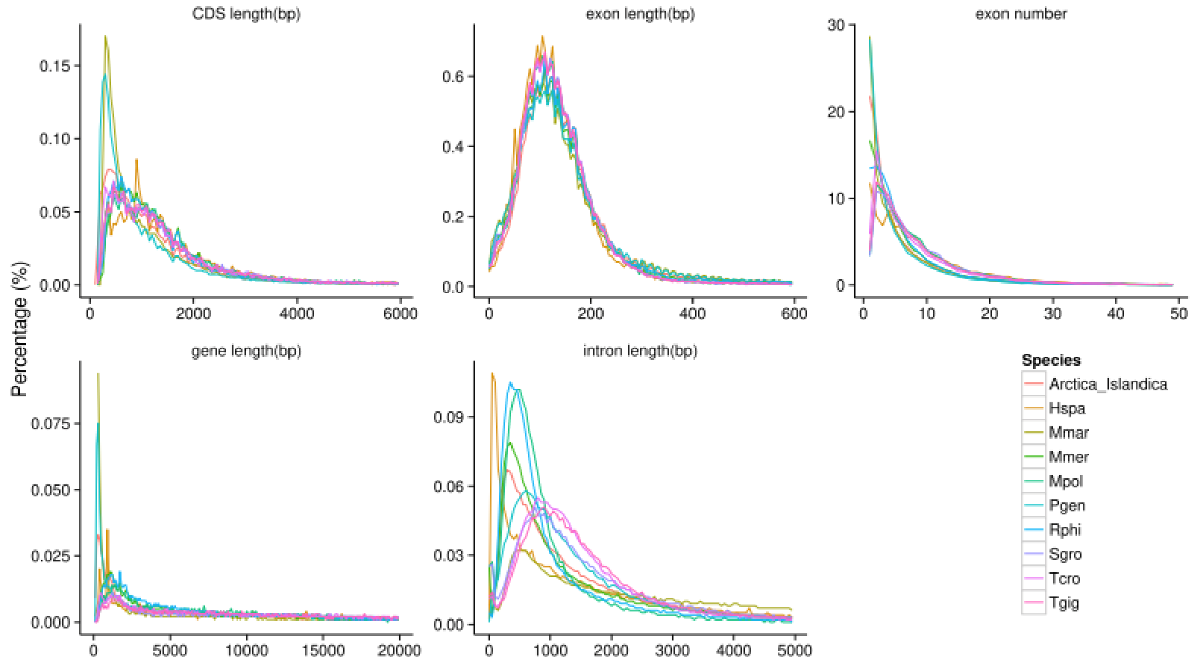

Figure S42. Gene structure length species comparison line chart.

Table S14. Final non-redundant gene set.

Gene set	Number	Average transcript length (bp)	Average CDS length (bp)	Average exons per gene	Average exon length (bp)	Average intron length (bp)
**EVM**	56,772	12,274.16	1,119.76	4.91	228.13	2,853.97
**Pasa-update***	56,461	12,565.54	1,136.81	4.99	227.67	2,862.09
**Final set***	39,509	15,429.13	1,326.67	6.03	220.12	2,805.34

* UTR included. Others: UTR not included.

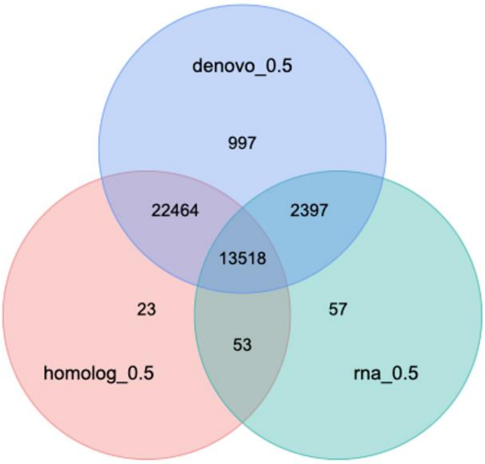

Figure S43. Venn diagram of gene set evidence support.*

*Note: de novo, genes supported by de novo prediction integrated by EVM; homolog, genes supported by homologous prediction integrated by EVM; RNA, genes supported by RNA-seq integrated by EVM. Evidence support is counted in when the location of corresponding prediction overlaps > 50% with the gene in the final gene set. Numbers indicate gene quantity.

Method S6.8. Gene function annotation

The gene set after gene structure annotation was aligned with known protein databases including SwissProt (http://www.uniprot.org/), Nr (http://www.ncbi.nlm.nih.gov/protein), Pfam (http://pfam.xfam.org/), KEGG (http://www.genome.jp/kegg/) and InterPro (https://www.ebi.ac.uk/interpro/) to generate gene function information. Protein sequences predicted by gene structure were aligned with known protein databases. Results show that the function of 98.3% of the genes could be predicted, as shown in Table S15.

Table S15. Proteins predicted by *Arctica islandica* gene structure aligned with known proteins in various databases.

**Database**	**Number**	**Percent (%)**
**Swissprot**	24,114	61.00
**Nr**	38,427	97.30
**KEGG**	27,367	69.30
**InterPro**	33,942	85.90
**GO**	18,335	46.40
**Pfam**	23,792	60.20
**Annotated**	38,850	98.30

**Unannotated**	659	1.70
**Total**	39,509	100

Method S6.9. ncRNA annotation

Non-coding RNA, or ncRNA, i.e., RNA that will not be translated to proteins, includes tRNA, rRNA, miRNA and snRNA. tRNA and rRNA are involved in protein synthesis. snRNA is a component of RNA spliceosome and is involved in RNA precursor processing. miRNA functions in RNA silencing and post-transcriptional regulation of gene expression. tRNAs were found by tRNAscan-SE (http://lowelab.ucsc.edu/tRNAscan-SE/) based on structural features. rRNAs were found by blast-based comparison to rRNA sequences of close species due to high conservation. miRNA/snRNA were predicted by INFERNAL (http://infernal.janelia.org/, provided by Rfam) based on covariance models (CMs).

Method S7. Versions of Genome Assembly Tools

Genome assembly assessment tool	Versions
wtdbg	2.5
FALCON	1.8.1
CANU	2.0
Nextdenovo	2.2
Hifiasm	0.8-dirty-r280
HiCanu	Canu branch V2.0
Smartdenovo	1.0
pilon	1.22
Racon	V1.3.1
Arrow	Smartlink8.0
Nextpolish	1.5
Bionano Solve	V3.5
LACHESIS	201701
ALLHIC	0.9.8
BUSCO	V4.1.2
CGEMA	V2.5
LTR_retriever	V2.9.0
BWA	0.7.8
Samtools	0.1.19
